# Adjusting for unmeasured confounding in nonrandomized longitudinal studies: a methodological review

**DOI:** 10.1016/j.jclinepi.2017.04.022

**Published:** 2017-07

**Authors:** Adam J. Streeter, Nan Xuan Lin, Louise Crathorne, Marcela Haasova, Christopher Hyde, David Melzer, William E. Henley

**Affiliations:** a Health Statistics Group, Institute of Health Research, University of Exeter Medical School, University of Exeter, St. Luke's Campus, Exeter EX1 2LU, United Kingdom; b Medical Statistics, Institute of Translational and Stratified Medicine, Plymouth University Peninsula School of Medicine & Dentistry, University of Plymouth, Plymouth Science Park, Derriford, Plymouth PL6 8BX, United Kingdom; c Mathematics, Physics & Electrical Engineering, Northumbria University, Sutherland Building, Newcastle upon Tyne NE1 8ST, United Kingdom; d Health Economics, Institute of Health Research, University of Exeter Medical School, University of Exeter, St. Luke's Campus, Exeter EX1 2LU, United Kingdom; e Evidence Synthesis & Modelling for Health Improvement, Institute of Health Research, University of Exeter Medical School, University of Exeter, St. Luke's Campus, Exeter EX1 2LU, United Kingdom; f Peninsula Technology Assessment Group, Institute of Health Research, University of Exeter Medical School, University of Exeter, St. Luke's Campus, Exeter EX1 2LU, United Kingdom; g Epidemiology & Public Health, RD&E Hospital Wonford, University of Exeter Medical School, RILD Building, Barrack Road, Exeter EX2 5DW, United Kingdom

**Keywords:** Method review, Unmeasured confounding, Unobserved confounding, Longitudinal, Observational data, Electronic health records

## Abstract

**Objectives:**

Motivated by recent calls to use electronic health records for research, we reviewed the application and development of methods for addressing the bias from unmeasured confounding in longitudinal data.

**Study Design and Setting:**

Methodological review of existing literature. We searched MEDLINE and EMBASE for articles addressing the threat to causal inference from unmeasured confounding in nonrandomized longitudinal health data through quasi-experimental analysis.

**Results:**

Among the 121 studies included for review, 84 used instrumental variable analysis (IVA), of which 36 used lagged or historical instruments. Difference-in-differences (DiD) and fixed effects (FE) models were found in 29 studies. Five of these combined IVA with DiD or FE to try to mitigate for time-dependent confounding. Other less frequently used methods included prior event rate ratio adjustment, regression discontinuity nested within pre-post studies, propensity score calibration, perturbation analysis, and negative control outcomes.

**Conclusion:**

Well-established econometric methods such as DiD and IVA are commonly used to address unmeasured confounding in nonrandomized longitudinal studies, but researchers often fail to take full advantage of available longitudinal information. A range of promising new methods have been developed, but further studies are needed to understand their relative performance in different contexts before they can be recommended for widespread use.

**What is new?**

**Key findings**
• Longitudinal information that can be used to mitigate for unmeasured confounding in observational data is not always fully or properly used in health research.
• Instrumental variable analysis and difference-in-differences were the most commonly encountered methods to adjust for unmeasured confounding in a review of the health literature.
• There are a range of promising new methods, some of which use longitudinal information to relax the assumption of time-invariance for unmeasured confounders, but these are yet to be widely adopted.

**What this study adds to what was known?**
• Unmeasured confounding is a threat to the validity of observational studies and this review highlights the potentially important role of longitudinal information in addressing confounding bias.

**What is the implication and what should change now?**
• All available methods for addressing unmeasured confounding rely on strong assumptions, and more research is needed to establish the relative performance of different methods for particular problems and empirical settings.

## 1 Introduction

In the era of “big data” in medicine, the increasing availability of large, longitudinal patient databases is creating new opportunities for health researchers. A particular focus is on electronic health records (EHR) with routinely collected data collated from multiple care sites, often linked to external databases (e.g., death certificates). Built up over time, EHRs provide a sequential history of each patient's encounter with the health care system. Examples of EHRs include The Clinical Practice Research Datalink, The Health Improvement Network, QResearch and ResearchOne in the UK, and the Kaiser Permanente Northern California Oracle Research Database in the United States. The value of large medical data recorded for administrative purposes in national registries is already recognized [1], [2], with the provision of funds to expand the adoption of EHRs in research for patient benefit with the Health Information Technology for Economic and Clinical Health Act of 2009 in the United States, and in the UK, with a consortium of funding bodies led by the Medical Research Council. Another important source of information for health care analysis is databases of insurance claims, such as Medicare in the United States, and in this review, we do not differentiate between EHRs and claims data.

A strength of EHRs and claims data is that they make it possible to study the comparative effectiveness of interventions and the associated risk of side effects in a real-world setting. Although randomized trials provide the gold standard of evidence, observational studies based on observational patient databases offer the potential to study more patients from a wider variety of risk groups with a longer follow-up period at a fraction of the cost. However, in the absence of randomization, selection for treatment is often knowingly based on specific characteristics, such as frailty, disease severity, or the risk of an outcome. If the indication for treatment is also related to prognosis, confounding by indication arises leading to biased estimation of effectiveness. There is a large pharmacoepidemiologic literature on this topic, and current best practice is to use design-based approaches such as the Active Comparator, New User Design to help mitigate bias where possible [3]. However, residual differences between the treatment arms other than the treatment itself may still confound the intervention effect under study whether or not such an approach is used. If the confounding variables are both known to the study investigators and measurable, then these could potentially be adjusted for in prospective nonrandomized studies. With retrospectively recruited subjects, however, the recording of such variables is outside the control of the investigator. Analyses of nonrandomized studies that fail to account for relevant confounders may have important negative consequences for health policy and patient safety.

Methods described as the quasi-experimental (QE) approach [4] can be deployed to account for confounding by unobservable characteristics. These do not attempt to directly adjust for resulting bias but use available information to achieve this indirectly under certain conditions and assumptions. The aim of this systematic review is to review current practices in dealing with unmeasured confounding in individual-level longitudinal health data and to capture methodological developments in this area. Although previous systematic reviews have been conducted to look at use of propensity score (PS) methods for measured confounders [5], [6], we are unaware of any systematic review comparing use of methods for addressing unmeasured confounding in nonrandomized longitudinal data. We were particularly interested in how an individual's history could be leveraged to evaluate the effects of unmeasured confounding and how the extra longitudinal information could be incorporated to improve adjustment for confounding bias. We intend for this review to contribute to the development of best practice in addressing unmeasured confounding in longitudinal data. The results should therefore help inform researchers intending to use “big data” from EHRs.

## 2 Methods

### 2.1 Search strategy

Our search strategy was informed by, but not limited to, known methods for addressing unmeasured confounding. The search strategy is recorded in Appendix A at www.jclinepi.com. The following electronic databases were searched: MEDLINE (via OvidSp including In-Process & Other Non-Indexed Citations) and EMBASE (via OvidSp 1996 to 2015 Week 21). We included all citation dates from database inception to May 2015. All references were exported into Endnote X7 (Thomson Reuters).

### 2.2 Inclusion and exclusion criteria

The review included any nonrandomized comparative studies that sought to adjust for unmeasured confounding in longitudinal data with repeated observations on identifiable individuals. In the interests of good practice, eligible papers had to explicitly identify the problem of bias arising from the selection on unobservable characteristics in the data, rather than routinely apply a QE design without this justification. For estimates of comparative effectiveness, eligible studies had to have independent control arms for each treatment of interest. Therefore, single-arm studies were excluded. Studies based on case-only designs, including the case-crossover design and the self-controlled case-series design, in which confounding is controlled by making comparisons between exposed and unexposed periods for the same individual were also excluded. Observational studies were not excluded based on the exposure under study so studies into the effects of passive exposures (medical conditions, environmental exposures, etc.) were included alongside studies of both the intended and adverse effects of active interventions. We note that good proxies for unmeasured confounding, or observed variables that sufficiently describe a latent variable such as frailty, would be preferable to dealing with the bias resulting from unmeasured confounders. If suitable proxies are identified and recorded, then there are in effect no unobserved confounders and the proxies could simply be adjusted for in the analysis, obviating the need for methods to adjust for the unobserved confounders. For this reason, adjustments for proxies of unmeasured confounders, including high-dimensional PSs, did not fall within the scope of this study. To be consistent with the “big data” theme of EHRs, a minimum sample size of 1,000 participants was applied. This also sets a minimum condition for the application of instrumental variable (IV) and regression discontinuity (RD) designs stipulated in the Quality of Effectiveness Estimates from Nonrandomized Studies checklist. Finally, we only accepted analyses of individual-level data. We were aware that some studies may use analytical methods such as difference-in-differences (DiDs) that aggregate the data at a treatment-group level. We therefore only included those studies in which the same patients could be tracked over the time frame of the sample. Conversely, some methods, such as instrumental variable analysis (IVA), make no explicit demands for longitudinal data at the patient level. However, we included such studies where the sample was based on the availability of patient-level longitudinal information, with a history possibly but not necessarily preceding the time of exposure. We did not discriminate between data sources, as patient-level data will often arise from medical insurance claims in the United States, as opposed to clinically purposed databases in other countries.

Only studies written in English were included.

The following publication types were excluded from the review:

• Systematic reviews of primary studies.
• Randomized controlled trials
• Cross-sectional data
• Preclinical and biological studies
• Narrative reviews, editorials, opinions

### 2.3 Study selection

Studies retrieved from the searches were selected for inclusion through a two-stage process according to the inclusion/exclusion criteria specified above. First, abstracts and titles returned by the search strategy were screened for inclusion independently by two researchers. In case of doubt, the article in question was obtained and a subsequent judgment on relevance was based on the full article. Disagreements were resolved by discussion, with involvement of a third reviewer when necessary. Following the initial screening, full texts of identified studies were obtained and screened firstly by a single reviewer. In case of doubt, a second reviewer decided on the suitability of a paper. Where multiple publications of the same study were identified, data were extracted and reported as a single study.

### 2.4 Evidence synthesis

The details of each study's design and methodology and the key characteristics of the data source were tabulated and discussed. We present a summary of the methods we found that can mitigate for confounding or its synonyms as unmeasured, unobserved, hidden, or residual. We note the historical frequency and context of the application of those methods, to comment on progress in causal inference and identify directions for future research.

## 3 Results

### 3.1 Included studies

**Fig. 1:**
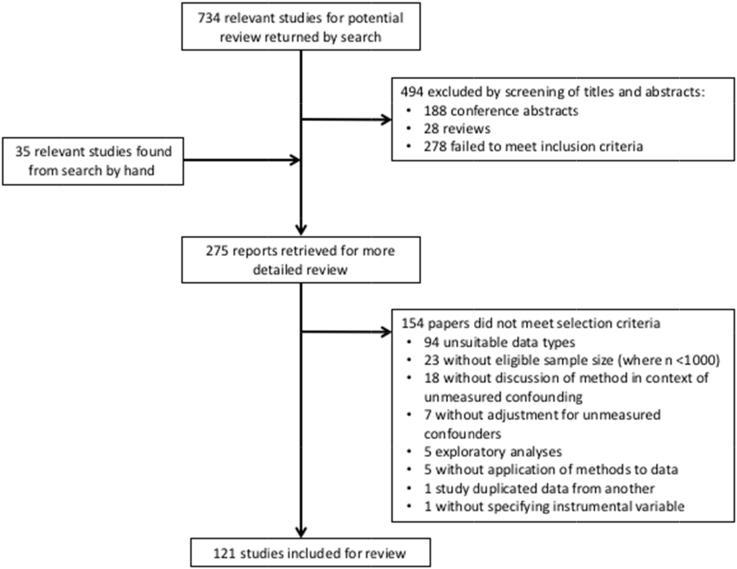
Flow diagram for method review.

Our searches returned 734 unique titles and abstracts, with 275 papers retrieved for detailed consideration. Of the 275 studies eligible for a full-text review, 154 were excluded (see flow diagram: Fig. 1).

A total of 121 studies were identified as performing a QE analysis on nonrandomized longitudinal data on human subjects, identifiable at an individual level, and so included for a full review of the text (Appendix B at www.jclinepi.com).

**Fig. 2:**
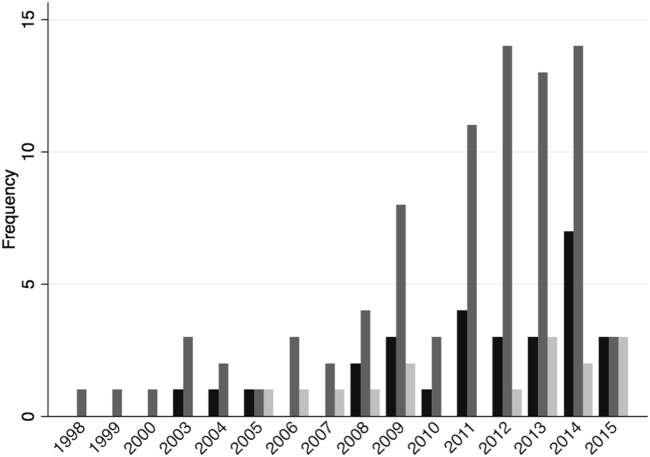
Plot of frequency of reviewed methods for mitigating for unmeasured confounding by: difference-in-differences (black); instrumental variable analysis (IVA) (mid-gray); other (light gray) includes regression discontinuity, prior event rate ratio method, propensity score calibration, perturbation analysis, negative control outcomes, fixed effects with IVA, and dynamic panel models. Note: the low frequencies in 2015 were attributable to the May cutoff for inclusion in that year.

The QE methods identified in the review are summarized in Table 1. The most frequent method was IVA found in 86 of the studies (Fig. 2)—a method that uses an unconfounded proxy for the intervention or exposure. For successful adjustment, the proxy or instrument should be strongly, causally associated with the exposure or intervention, and the instrument should only affect the outcome through the exposure. In addition to IVA, three of these also applied DiDs—a method that typically uses pre-exposure outcomes to adjust for unmeasured confounding and assumes any trends unrelated to the exposure—are the same in both groups. Seven more studies derived estimates from a combination of both IVA and DiD, two of which assumed an absence of higher order autocorrelation to use lagged observations of the treatment variable as an instrument. Beside the 10 studies applying DiD either in conjunction with or in addition to IVA, we identified further 21 studies, in which the sole QE method was recognized as a DiD approach.

**Table 1:** Summary of methods to mitigate against unmeasured confounding captured by systematic review and the frequency of their use among the captured papers

Method	Description	Obstacles to implementation	Frequency of methods
Instrumental variable analysis (IVA)	Upon identification of a suitably strong instrument, the influence of bias may be reduced through post hoc randomization. The instrumental variable should be highly determinant of the intervention or treatment received, while satisfying the exclusion assumption of being independent of the outcome other than through the treatment (Wright 1928; Angrist 1991).	In practice, finding an instrument with a sufficiently strong treatment association is a stumbling block in many analyses (Bound, Jaeger, and Baker 1995; Baser 2009). Association of the instrument with the outcome exclusively through the treatment is an untestable assumption, particularly if an indirect association exists through an unmeasured covariate.	79
Difference in differences (DiDs)	A biased effect estimate between two treatment groups may be corrected by the same estimates from a treatment-free period before the exposure, which should be a measure of the confounding bias contributed to the treatment effect (Ashenfelter and Card 1984). Aggregated at the treatment group level, this is operationalized in regression as a period-treatment interaction. At an individual level, demeaning, first-differencing or dummy variables for each individual may yield bias-free fixed effects, contingent on assumptions.	The method is contingent on the availability of repeated outcomes in both periods and invokes a time-invariant confounding assumption: that the confounding bias as captured by the estimated treatment effect in a treatment-free period before exposure is constant through to the study period.	24
Prior event rate ratio (PERR)	Analogous to the DiD method for time to event or rate data, a biased estimate of the hazard ratio or the incidence rate ratio is adjusted through its ratio with that from a treatment-free prior period (Tannen et al. 2008).	As with the assumption for DiD, repeatable outcomes and a constancy of the unmeasured confounding bias are required across both periods, before and after the exposure. Prior event occurrence should not influence the likelihood of future treatment.	5
Fixed effects instrumental variable analysis (FE IVA)	IVA may be applied to DiD estimation to mitigate for the second-order endogeneity: the time-varying part of the bias that may not have been adjusted for by DiD.	Assumptions of IVA apply	5
Dynamic panel model or instrumental variable—generalized method of moments (IV-GMM)	Lagged observations of the confounded (endogenous) explanatory variable are introduced in a first-differences fixed effects analysis so that the differences of the lags become the instrumental variables in a generalized method of moments estimation.	Assumptions of IVA apply. Here, the differenced lags should not be correlated with the differences in the error terms.	2
Regression discontinuity (RD)	RD is a design for analysis based on a treatment assignment determined by a cutoff applied to a continuous variable, that is, preferably measured with some random noise (as many clinical tests may be). The outcome can then be modeled on treatment for individuals within a certain interval from the cut-off of the assignment variable to ensure exchangeability between individuals for robust causal inference (Thistlethwaite and Campbell 1960)	Where assignment is not sharply determined by the cutoff, an increase in the probability of treatment may be observed leading to a “fuzzy” version of RD. Continuity in the assignment variable is assumed, otherwise, manipulation of assignment, and reverse causality may be suspected. Assignment should be locally random around the cutoff and makes the weak assumption that no unobserved covariates are discontinuous around the assignment cutoff.	3
Propensity score calibration (PSC)	PSC adjusts for residual confounding in the error-prone main data set by importing information about the unmeasured confounders from a smaller, external “gold-standard” data set (Stürmer et al. 2005). Analysis in the main data set is adjusted using a single-dimension propensity score of the measured corrected for unmeasured confounding by regression calibration against the gold-standard propensity score.	Successful adjustment is wholly dependent on the availability of another data set containing the exposure variable and error-free predictor, with individuals that are relevant enough to those in the main data set and under similar enough conditions to assure sufficient overlap between the two data sets.	3
Perturbation testing/analysis (PT/PA)	This data mining approach aims to mitigate for unmeasured confounding by adjusting for many measured variables that are weakly associated with the unobserved confounding variables (Lee 2014). Simulation in the single-reviewed example demonstrated this may require 100s, if not 1000s of perturbation variables (PVs).	This requires a very highly dimensional data set, which may ultimately obviate the need for indirect adjustment if the most or all of the confounders are captured. Simulation demonstrated that the bias may be exaggerated if a confounder is inadvertently identified as a PV, requiring many more true PVs to correct the bias. The number of PVs may exceed the available degrees of freedom necessitating clustering.	1
Negative control outcome/exposure (NCO/NCE)	A negative control is causally related to measured and unmeasured confounders affecting the exposure and main outcome but not directly causally related to exposure and outcome themselves. As such, the negative control may be used to detect confounding bias in the main study and potentially to indirectly adjust for this (Richardson et al. 2014)	This assumes that the effect of the unmeasured confounders on the main outcome is similar to that affecting the negative control.	1

We found five studies applied the prior event rate ratio method, a before-and-after approach, that can be aggregated to the treatment level for survival or rate outcomes and analogous to DiD. In all five cases, the methods were applied to longitudinal individual patient data. Similarly, RD was used for such data in three of the studies included for review. Another three focused on propensity score calibration (PSC). One study introduced perturbation testing and perturbation analysis (PA), whereas another discussed the use of negative control outcomes.

#### 3.1.1 Studies excluded at full text

The principal reason for exclusion in 94 of the studies, according to our eligibility criteria, was the absence of longitudinally observed, nonrandomized outcomes on all individually identifiable persons, although other characteristics may also have justified their exclusion. No particular method was associated with the absence of longitudinal data on identifiable individuals with the studies in this exclusion category comprising 59% DiD and 28% IVAs compared, respectively, to 53% and 32% of all 154 of the rejected studies. Having fewer than 1,000 longitudinally observed individuals excluded 23 studies, among which those using IVA numbered 15. Seven were excluded for not using a QE method for unmeasured confounding. Five studies presented exploratory analyses without a focused clinical question; five were either method reviews or commentaries without an application of methods to data; one study duplicated a data set already marked for inclusion, whereas another failed to specify the IV used. Of particular note were the 18 studies using the DiD approach that were excluded because no explicit justification was made for using the method to address unmeasured confounding or any of its synonyms. In these studies, justification of the method was centered more on econometric concerns over time trends and presented in terms of controlling for those trends rather than pre-existing differences between the control and exposed group.

### 3.2 Results of the included studies

So far studies have been categorized according to their identified QE method. However, certain properties are shared across some of the methods and can be classified according to how they reconcile their specific assumptions with the information offered by the structure of big, longitudinal data that typifies EHRs. In particular, we organized our results around how each method had incorporated longitudinal information, and the assumptions required. The stable of before-and-after methods, that includes PERR and DiD, implicitly incorporates longitudinal information. Thereafter, the challenge is how to relax the assumption of time-invariant confounding. Conversely, IVA is not uniquely applicable to longitudinal data, but we were able to broadly classify the types of instruments used (Table 2), some of which did use longitudinal information. We found out of the total 121 studies, 77 incorporated some element of longitudinal information into their analysis.

**Table 2:** Frequency of instruments categorized by type used in instrumental variable analyses

IV type	Explanation/example	Frequency
Historical	Usually prescribing preference of physician or facility based on historical records of previously administered therapies	34
Geographic	Differential distance between patient's postcode and nearest health facility	20
Mendelian	Genetic characteristics: single nucleotide polymorphisms	11
Time	Time-based characteristic of treatment such as date of therapy	10
Other	Characteristics of individual, for example, age of patient, weight of offspring	8
Lagged	Previous therapy or outcome of patient	6
Randomization	Original randomization	1

*Abbreviation:* IV, instrumental variable.

#### 3.2.1 Incorporation of external/additional data

Propensity scores (PS), the predicted probability of exposure or treatment conditioned on measured confounders, were used in the seminal work on PSC by Stürmer to calibrate an error-prone PS against a gold-standard PS and hence arrive at an inference for the level of unmeasured confounding bias [e1]. The two subsequent PSC papers examined the tenability of the method's assumptions, first using simulated data to evaluate the conditions necessary to violate the surrogacy assumption [e2]. The second primarily used simulated data and applied the results to registry data to demonstrate a framework for determining size and direction of bias from one measured and one hidden confounder [e3].

#### 3.2.2 High-dimensional data

Because PSC collapses multiple, potential confounding variables down to the single dimension of a PS, the three PSC papers can also be considered a means of dealing with high-dimensional data. In addition to these, our review also included a novel data-mining approach that proposed to exploit the many factors (perturbations) that may be weakly associated with the unmeasured confounders from a high dimension data set, [e4] for which longitudinal data may mitigate for incorrect adjustment of a collider. PA was successfully demonstrated on simulated data, although accidental inclusion of a measured confounder required many more perturbations to correct the resulting bias. Both the perturbation method and PSC were also proposed as sensitivity analyses.

#### 3.2.3 QE adjustment without longitudinal assumptions

Those studies characterized as using a QE method without any longitudinal dimension were PSC and PT as described above. We also added to this category 11 examples of Mendelian IVA [e5–e15] plus 32 other IVAs without historic or lagged instruments [e16–e47]. Although time-based instruments may at first seem longitudinal, these instruments, such as date of therapy, would need to be related to previous exposures or outcomes to be considered longitudinal. In some cases, survival times or rate data were used, but such outcomes do not intrinsically imply longitudinal adjustment for confounding. In spite of these “cross-sectional” approaches, all studies were based on some form of longitudinal data at the person level, as demanded by our inclusion criteria. Among the 43 non-Mendelian IVA papers in this nonlongitudinal category, one study adjusted for nonlongitudinal fixed effects (FEs) within twins [e33]. In another three, discussed below, the analysis was supplemented with DiD [e32,e41] and with IVA applied to first differences [e48].

One study examined the effect of lagged, cumulative exposure to radiation on lung cancer in uranium miners and nuclear workers [e49]. The problem of unmeasured confounding was addressed using a method developed in earlier work that proposed negative control outcomes and exposures as a means of both detecting and potentially resolving confounding bias [7]. Here, the choice of death due to chronic obstructive pulmonary disorder as a negative control outcome was informed by clinical knowledge of there being no direct relationship with the exposure except through the possible confounder, smoking. Given a plausible negative control outcome or exposure, the method offers at least a means of testing for confounding and potentially a method of adjustment under the assumption that the association between the unmeasured confounder and the negative outcome is similar in magnitude to that between the same confounder and the outcome of interest.

#### 3.2.4 QE adjustment assuming time-invariant longitudinal information

We found 37 IVA studies that used lagged information or history about the individuals' exposure as instruments [e48,e50–e85]. One study had recourse to the random assignment from a previous study and used this as an instrument [e62]. Except for that and four other different exceptions, the instruments were all based at least in part on the previous intervention, or history of interventions, of the clinician or health care facility. Characteristics of the clinician or facility may be chosen as instruments as they are more likely to affect the treatment only. This avoids direct associations with the individual and their outcome, and so, better enforces the exclusion restriction—the exclusion of the instrument's association with the outcome except through the treatment under study. Although no assumptions are made about the dependence of confounding on time, the strength of the instrument clearly rests on a significant association between previous treatment(s) and the current treatment under investigation. In this regard, if the strength of an instrument varies with time, this may undermine its utility.

In total, 24 studies also incorporated longitudinal information through the stable of methods that, in an abuse of terminology, we collectively referred to as the DiD approach. These included the 18 examples cited as using DiD regression [e86–e103] alone and four FEs [e104–e107]. Either through FEs at the individual level or through aggregate-level regression operationalizing the DiD approach, these methods “ignore” the effect of confounding, which is assumed to be time invariant. At the individual level, time-invariant confounding can be ignored by assigning nuisance dummy variables for each individual or canceled out through demeaning the observations or through the first differences of observations on each individual. Two of the studies also extended DiD to allow different exposure effects and trends across two-level subgroups in the higher-order contrast of difference-in-difference-in-differences [e88,e99]. Fourteen studies also adjusted for individual-level FEs either through direct inclusion of their covariates or through matching or weighting on the PS of the covariates. This was perhaps a more rigorous and precise approach, accounting for known confounders and yielding smaller standard errors for the estimated treatment effect. However, an assumption of time-invariant confounding was still required, with a null difference between exposure groups in the prior period being evidence of adjustment for time-invariant confounding only. Two of the 24 DiD studies also reanalyzed their data using IVA, [e32,e41] which provided an albeit limited opportunity to compare the relative performance of these methods. In the study by Schmittdiel et al. of how statins delivered by mail order affects cholesterol control, [e41] the intervention coefficient from modeling the single main outcome was larger through DiD analysis and its standard error smaller than those from IVA, large standard errors being a feature of weak instruments. The study by Lei and Lin investigated the effect of exposure to a new medical scheme on 15 health outcomes and rates of health-service utilization [e32]. The effects were either not significantly different from the null or were significant and of similar magnitude with similar standard error except for two outcomes, where the effect size was significantly larger for IVA.

Time-invariant confounding, also known as the parallel trends assumption, was relaxed by including dummy variables for the year and its interaction with the treatment dummy in a fixed-effects analysis, which allowed the unobserved trend to vary between exposure groups [e106] using methods developed in economics and therefore not captured by this review [8], [9]. The results from this DiD with differential trend model were presented alongside those from the simple pooled DiD model and DiD with individual fixed effects for the effect of financial incentives in care services. Tests confirmed parallel trends could be assumed in three outcomes, but out of the five outcomes presented, four were statistically significant, and in all, the estimated effect size by differential trends was greater.

Our review also included five studies applying the prior event rate ratio method, a before-and-after analogue applicable to survival and rate data [e108–e112]. The first two published were the seminal presentation of the method applied to registry data. Also included was a comprehensive evaluation by Uddin et al. of the performance of PERR under a wide array of simulated, theoretical settings, under which bias was shown to increase with a greater effect of the prior events on subsequent exposure or intervention. When prior events strongly influence the likelihood of treatment, the exposure effect from the PERR method can be more biased than estimates from conventional methods [e112]. The problem was re-examined in a recently published study, which provided a more general statistical framework for PERR adjustment and considered the potential for generalizing the method to allow more flexible modelling [10].

#### 3.2.5 Dynamic, longitudinal QE methods, and time-varying information

Although RD could suggest a longitudinal design, this is not exclusively so, and two RD studies were excluded because of this (one applied to spatial data, whereas the other data were not longitudinal). Of those included, all three could be said to accommodate time-varying trends, [e113–e115] and two of these were nested within a pre-post design: Zuckerman et al. were explicit in their methodological study in identifying the robustness to time-varying confounding, in which inhaler use in asthmatic patients was served as both the outcome variable in the posttest period as well as the assignment variable in the pretest period [e115]. In the study of the effect of school-leaving age on mortality by Albouy, different slopes were modeled for the assignment variable, year of birth, and after the cutoff date [e113]. This acknowledged different maturation rates after assignment. However, as long as the assumptions of the method were met, assignment should have been as good as randomized, and so, no further assumptions about the temporality of confounding was required.

We also picked up seven examples where IVA had been combined with either a DiD or a FEs model, first appearing in our review with an example from 2003 [e116]. In Fortney's 2005 study of treatment for depression, [e117] this combination method was justified as a control for time-varying confounding, referred to as second-order endogeneity. Further examples of the fixed-effects IV model were found [e118,e119]. The roles of lagged treatments and outcomes as possible IVs and predictors were extensively considered in O'Malley's study of whether the introduction of more expensive medication could have led to improved cost-effectiveness in the long term [e48]. The author cautioned that the exclusion restriction may be difficult to satisfy when using the lagged treatment as an IV after first differencing. However, two studies [e120,e121] used differences in the lagged explanatory variable as the IVs to adjust for second-order endogeneity in a first difference analysis following methods, not captured by our review, but developed in the realm of Economics [11], [12], [13]. Referred to as the dynamic panel model or IV-GMM, this method was implemented efficiently using the generalized method of moments. In their report on health care expenditure in patients with rheumatoid arthritis, Kawatkar et al. found the yielded estimates were further from the null with larger standard errors when compared to those from FE alone [e120].

### 3.3 Implementation of methods

Although choice of method in each study often rested on which extra information was available to address the issue of unmeasured confounding, method selection may also have been informed by the research area. The negative control method had its origins in epidemiology, with applications to occupational health policy. Likewise, the PERR method was developed exclusively on health data, with applications to drug safety and public health policy. Reflecting their origins in health econometrics, some studies that used DiD or IVA were published in journals partially or entirely dedicated to health economic evaluations, with 15 published [e32,e48,e86–e88,e91,e196,e97,e104–e107,e116,e117,e120] in this field out of the 32 studies using DiD and 29 [e17,e18,e22–e24,e26,e27,e30,e35,e40,e42,e43,e45,e46,e59,e62–e65,e70,e72,e74,e77,e79], out of the 86 using IVA. Under the inclusion criteria, all studies had health outcomes or interventions. Mendelian IVA necessarily includes genetic information, and all were published in health-related journals. In contrast, all three studies using RD were published in health econometric journals.

Before implementing one of the proposed methods, a natural first step is for the researcher to try to assess how much bias from unmeasured confounding is likely to be present. Although many of the included studies reported raw or unadjusted descriptive estimates, bias estimation was limited either to considering the contribution from known confounders, including those summarized as a PS, or to methods, such as perturbation testing/analysis and negative controls methods, in which bias evaluation is an incremental step in adjustment. Under the assumption of time-invariant confounding, the DiDs method may potentially offer a way of evaluating bias by modeling group differences in the pre-exposure period. However, few studies evaluated hidden bias in this way [e41,e89,e105]. The regression formulation of the DiD method effectively by-passes separate analysis of the prior period. Instead studies often discussed the within-group changes over time. Similarly, the prior-period estimate from the PERR method implicitly offers an evaluation of confounding bias under the same assumptions, yet none of the studies presented information on outcomes in the prior period in this way. A direct evaluation of unmeasured confounding is less straight-forward in IVA, with further diagnostic tests only recently developed for the association between instrument and confounders [14], [15].

## 4 Discussion

This review examined the application of methods to detect and adjust for unmeasured confounding in observational studies and was motivated by recent calls to use EHRs. Most of the reviewed studies used more established methods such as DiD and particularly IVA. We summarized how studies exploit the longitudinal information afforded by EHRs.

It may be tempting to view EHRs and medical insurance claims data as a problem of large observational data and hence search for solutions through data mining. However, ethics governing patient data collection plus limited clinician time is likely to preclude data with very large dimensions. For that reason, it is doubtful there would be enough dimensions for a method like PA to be a practical solution. In addition, a greater number of variables would likely include enough information about the confounders to obviate the need for further adjustment through PA. More generally, the purpose of EHRs primarily as an administrative tool limits the scope for data mining of known confounders. Similarly, limited availability of gold-standard data sets may have confined the use of external data, as in PSC, to but a few examples.

We were surprised by the number of studies using IVA alone. Although Mendelian randomization has its advantages for many studies as a reasonable guarantor of the exclusion restriction, in general, IVA typically suffers from the weak-instrument problem, resulting in large standard errors and wide confidence intervals. Longitudinal data offer an opportunity to reinforce the exclusion criteria by choosing historical or lagged instruments. However, in doing so, the causal structure needs to be understood to avoid opening up “back door” paths and inducing further bias [e48]. DiD arguably offers advantages over IVA in being more intuitive and easier to conceptualize, and with the longitudinal data in EHRs, it may often be easier to work with prior observations than to identify strong instruments. Even though before-and-after methods are not subject to the imprecision of weak instruments, the resulting estimates are only unbiased if the unobserved confounders exert a constant effect over the observation windows before and after exposure. Where multiple observations per individual exist, time may be parameterized and different trends between exposure groups can be accommodated in DiD with differential trends, but a time-invariant assumption about confounding must still be made. To partially or wholly relax this particular assumption, instrumental variable analysis can be incorporated into the FEs model. Assuming the instrument's exclusion restriction is satisfied, then this doubly robust approach affords the advantage of DiD over possibly weak instruments, while mitigating for some or all of the time-dependent confounding ignored by DiD alone. Similarly, where multiple previous treatments or exposures are recorded, the differenced lagged treatments can be used as IVs in a FEs model to accommodate time-dependent confounding bias using the generalized method of moments system, referred to as IV-GMM or the dynamic panel model.

Another potentially robust approach to unmeasured confounding would be the RD design, although the small number of examples in our review probably reflects the limited number of scenarios where this can be reasonably applied. Another concern over and above the usual technical challenges of applying the RD method is that in spite of heath records promising ample data, the sample would need to be reduced to an interval around the cutoff that ensures exchangeability of the two treatment groups. In this case, generalizability would be restricted to individuals with characteristics found in the interval. As with RD, PERR was another method that was found in relatively few studies. This may have been in large part due to its recent development, rather than any technically demanding aspect of its application because it simply extends the before-and-after approach of DiD to survival and rate data — outcomes that are common in health research. However, the PERR approach does require strong assumptions including time-invariant confounding and the absence of an effect of prior events on likelihood of future treatment [10].

Methods such as IVA and DiD have their origins in the sphere of econometrics, where randomized experiments are rare. We found that in importing DiD, some of the studies failed to explicitly acknowledge the problem of confounding bias. Instead justification for the method was presented in terms of the common trends assumption. Discussion of possible confounding bias is regarded as essential by most QA toolkits for observational data, and it is important that health researchers explicitly recognize this threat to the internal validity of nonrandomized studies. Conceptually, a nontemporal analogue of DiD would be the NCO method, which itself was presented foremost as a method for detecting unmeasured confounding. Given doubts over satisfying necessary assumptions for their implementation, authors of this method along with PSC and PA have suggested that, as sensitivity analyses, these can at least offer an insightful complement to QE adjustment.

Choosing between methods to reduce unmeasured confounding bias is challenging, and we found few studies that directly compare methods. The performance of different methods will depend on factors such as the nature of the underlying confounding, the type of exposure and outcome, and the sample size [16]. The type of data available will also guide the choice of method. For example, the IV method requires a suitable instrument, and DiD/PERR requires data on at least two periods. In practice, no one method is likely to be best suited to all problems, and it is essential for investigators to carefully assess the potential biases in each proposed study, where possible tailoring the methods or combination of methods to address these biases [17]. Our review has highlighted how use of longitudinal information is one additional and potentially important consideration in this process.

Although our review focused on the problem of adjustment using analytic methods, many problems associated with observational data may be pre-empted by use of an appropriate study design [18]. Before choosing an appropriate analytic method, it is recommended that investigators carefully identify and match individuals for the control and intervention groups in order not to exacerbate any bias [3]. The importance of study design is often discussed with a view to minimizing confounding bias from unmeasured sources, with the subsequent adjustment accounting for observed confounders only [19], usually through the matching, weighting, or adjustment of PSs [20]. Where the success of the design remains in doubt or its criteria cannot be fully met, then investigators will inevitably need recourse to some of the alternative methods reviewed in this report.

The reviewed studies did not seek to distinguish between the different mechanisms of bias. Confounding by indication, deemed intractable by many researchers using the observed data [21] was seen to create additional sources of bias in two separate simulation studies applying the “longitudinal” method of PERR, when an association was modeled between prior events and treatment status in the study period [e112,10]. Another common form of selection bias in pharmacoepidemiologic studies is the healthy user bias, and this works in the opposite direction to confounding by indication, distorting treatment-outcome associations toward the treatment looking beneficial [3]. Further research is needed to understand how each of the methods in this review is affected by the different types of confounding.

An inherent limitation of this large, wide-ranging review is that it precluded meaningful data synthesis due to the mix of different data and study types. Furthermore, we could only find a few examples where the performance of different methods was compared within the same study. We also stipulated in the inclusion criteria that unmeasured confounding, or any of its synonyms, should be given as justification for using the identified methods for QE adjustment. This may have inadvertently excluded some papers, where justification was implicit, but good practice in health research demands acknowledgment of this source of bias where applicable. Although our search terms were specific to the scope of our review, we accept that this may have inadvertently excluded relevant methods and studies. Some methods, such as negative control outcomes, that were identified in the original search were not included as explicit terms in the search strategy, and further secondary searches may have uncovered additional studies using these methods. We also acknowledge that there may be other relevant methods for addressing unmeasured confounding that have been missed by the search strategy. Consequently, we made inferences about the relative application of methods with caution. However, we were surprised so many studies focused solely on IVA as the sole means of adjustment. A similar conclusion was echoed by a different review on RD designs that found interest was growing in RD only as recently as 2014 [22].

By choosing to focus on methods with an independent control arm for each treatment, our review excluded case only designs including case-crossover (CCO) designs and the self-controlled case-series design. This class of methods addresses unmeasured confounding by making comparisons within individuals so that each individual acts as his or her own control. Another case-only design, the case-time control design, is an extension of the CCO design that uses information from a historical control group in a similar way to the PERR method. These approaches are reviewed by Uddin et al. [16] and Nordmann et al. [23].

This review has considered a range of promising new methods for addressing unmeasured confounding in nonrandomized studies. However, consistent with prior research on dissemination and uptake of statistical innovations [24], the rate of knowledge translation has been slow and we found that most studies in our review used established methods such as IVA and DiD. A recent study by Cadarette et al. has shown how Rogers’ Diffusion of Innovations model can be used to describe the adoption of novel methodologies in pharmacoepidemiology [25], and this provides a useful resource for interpreting the uptake of methods in this review. Cadarette et al. proposed five principles for authors of methodological innovations that may improve translation into practice [25]: (1) clearly describing the methods using foundational principles; (2) comparing results to established methods; (3) providing sample data, code, or calculation examples; (4) early communication, support, and testing; and (5) providing methodological and reporting guidance. These recommendations offer a useful checklist for researchers developing methods for addressing unmeasured confounding in observational studies. Of particular relevance in the context of this review is the need for more extensive evaluation and comparison of the emerging methods in a range of settings. The review also addresses the need for methodological guidance through highlighting the potentially important role of longitudinal information in addressing confounding bias and has identified this as an area for further development.

## 5 Conclusions

Our review showed how seminal work in econometrics has influenced practice in dealing with unmeasured confounding in clinical and epidemiological research. Although the issue of unmeasured confounding is widely acknowledged, we found that longitudinal information in observational studies appears underused. Lagged and historical characteristics associated with the treatment may help enforce the exclusion restrictions of IVs under the appropriate causal structures, while before-and-after methods, such as DiD and PERR, afford an intuitive approach without the imprecision of weak instruments. Furthermore, they offer a direct evaluation of time-invariant confounding bias. The most robust methods we found applied IVA to the FEs DiDs method, where suitable instruments or difference lagged variables could be assumed to satisfy the exclusion restriction. Although there are sometimes good technical reasons for choosing one mode of analysis over another, many questions remain over the most appropriate methods. All methods rely on assumptions, but little guidance is available to applied researchers as to the empirical settings in which particular methods can be safely used. Few studies directly compare different methods, and more research is needed to establish the relative performance of the methods in realistic settings.

## Supplementary data

Appendix B

Supplementary References
