# Supplementary material for: Adjusting for unmeasured confounding in nonrandomized longitudinal studies: a methodological review
Source: J Clin Epidemiol. 2017 Jul;87:23–34. doi: 10.1016/j.jclinepi.2017.04.022 (PMC5589113; doi:10.1016/j.jclinepi.2017.04.022)
Supplement: Appendix A [file mmc1.docx]

# Appendix A

1. ("prior event" and ratio).ti,ab.

|  |
| --- |

2. "paired cox model".ti,ab.

|  |
| --- |

3. 1 or 2

|  |
| --- |

4. instrumental variables.ti,ab.

|  |
| --- |

5. instrumental variable analysis/

|  |
| --- |

6. propensity score calibration.ti,ab.

|  |
| --- |

7. regression discontinuity design.ti,ab.

|  |
| --- |

8. "difference in differences".ti,ab.

|  |
| --- |

9. (difference adj1 differences).ti,ab.

|  |
| --- |

10. "ratio of ratios".ti,ab.

|  |
| --- |

11. (ratio adj1 ratios).ti,ab.

|  |
| --- |

12. interrupted time series.ti,ab.

|  |
| --- |

13. segmented regression.ti,ab.

|  |
| --- |

14. (sensitivity analysis/ or sensitivity analysis.ti,ab.) and ((unmeasured or residual or hidden) and (confounding or confounder*)).ti,ab.

|  |
| --- |

15. or/4-14

|  |
| --- |

16. ((unmeasured or residual or hidden or unobserved or omitted) and (confounding or confounder*)).ti,ab.

|  |
| --- |

17. confounding variable/

|  |
| --- |

18. covariates.ti,ab.

|  |
| --- |

19. bias.ti,ab.

|  |
| --- |

20. selection bias/

|  |
| --- |

21. 16 or 17 or 18 or 19 or 20

|  |
| --- |

22. observational study/

|  |
| --- |

23. (observation* adj (stud* or data)).ti,ab.

|  |
| --- |

24. ((before adj after) and (study or studies)).ti,ab.

|  |
| --- |

25. (nonrandomi?ed or non randomi?ed).ti,ab.

|  |
| --- |

26. case crossover.ti,ab.

|  |
| --- |

27. case control.ti,ab.

|  |
| --- |

28. case control study/

|  |
| --- |

29. cohort study.ti,ab.

|  |
| --- |

30. (quasi experiment* or quasiexperiment*).ti,ab.

|  |
| --- |

31. quasi-experimental study/

|  |
| --- |

32. cross sectional study.ti,ab.

|  |
| --- |

33. cross-sectional study/

|  |
| --- |

34. simulation.ti,ab.

|  |
| --- |

35. case time control.ti,ab.

|  |
| --- |

36. ("before and after" and (study or studies)).ti,ab.

|  |
| --- |

37. or/22-36

|  |
| --- |

38. 16 and 19 and 37

|  |
| --- |

39. 3 or 15

|  |
| --- |

40. 39 and 37 and 21

|  |
| --- |

41. 38 or 40

|  |
| --- |

42. 21 or 37

|  |
| --- |

43. 39 and 42
