# Supplementary material for: Adjusting for unmeasured confounding in nonrandomized longitudinal studies: a methodological review
Source: J Clin Epidemiol. 2017 Jul;87:23–34. doi: 10.1016/j.jclinepi.2017.04.022 (PMC5589113; doi:10.1016/j.jclinepi.2017.04.022)
Supplement: Appendix B [file mmc2.docx]

# Appendix B

Table 3: Table of included studies denoting QE method used and type of instrument, if applicable, where: IVA = instrumental variable analysis; RD = regression discontinuity; DiD = difference-in-differences; DiDiD = difference-in-difference-in-differences; PSC = propensity score calibration; PERR = prior event rate ratio

| Author | Title | Year | QE method | If IVA, IV type |
| --- | --- | --- | --- | --- |
| Bryson, W. C.; McConnell, J.; Krothuis, T.; McCarty, D. | Extended-release naltrexone for alcohol dependence: persistence and healthcare costs and utilization | 2011 | DiD |  |
|  |  |  |  |  |
| Cheng, L.; Liu, H.; Zhang, Y.; Shen, K.; Zeng, Y. | The impact of health insurance on health outcomes and spending of the elderly: Evidence from china's new cooperative medical scheme | 2015 | DiD |  |
| Gebel, M.; Vosemer, J. | The impact of employment transitions on health in Germany. A difference-in-differences propensity score matching approach | 2014 | DiD |  |
| Goetzel, R. Z.; Roemer, E. C.; Pei, X.; Short, M. E.; Tabrizi, M. J.; Wilson, M. G.; Dejoy, D. M.; Craun, B. A.; Tully, K. J.; White, J. M.; Baase, C. M. | Second-year results of an obesity prevention program at the dow chemical company | 2010 | DiD |  |
| Higgins, S.; Chawla, R.; Colombo, C.; Snyder, R.; Nigam, S. | Medical homes and cost and utilization among high-risk patients | 2014 | DiD |  |
| Kausto, J.; Viikari-Juntura, E.; Virta, L. J.; Gould, R.; Koskinen, A.; Solovieva, S. | Effectiveness of new legislation on partial sickness benefit on work participation: a quasi-experiment in Finland | 2014 | DiD |  |
| Kelly, Y.; Kelly, J.; Sacker, A. | Changes in bedtime schedules and behavioral difficulties in 7 year old children | 2013 | DiD |  |
| Lin, W. C.; Chien, H. L.; Willis, G.; O'Connell, E.; Rennie, K. S.; Bottella, H. M.; Ferris, T. G. | The effect of a telephone-based health coaching disease management program on medicaid members with chronic conditions | 2012 | DiD |  |
| Lyon, S. M.; Wunsch, H.; Asch, D. A.; Carr, B. G.; Kahn, J. M.; Cooke, C. R. | Use of intensive care services and associated hospital mortality after massachusetts healthcare reform | 2014 | DiD |  |
| Menon, J.; Paulet, M.; Thomas, Iii J. | Wellness coaching and health-related quality of life: A case-control difference-in-differences analysis | 2012 | DiD |  |
| Moran, J. R.; Short, P. F.; Hollenbeak, C. S. | Long-term employment effects of surviving cancer | 2011 | DiD |  |
| Osborne, N. H.; Nicholas, L. H.; Ryan, A. M.; Thumma, J. R.; Dimick, J. B. | Association of hospital participation in a quality reporting program with surgical outcomes and expenditures for medicare beneficiaries | 2015 | DiD |  |
| Reid, R. O.; Ashwood, J. S.; Friedberg, M. W.; Weber, E. S.; Setodji, C. M.; Mehrotra, A. | Retail clinic visits and receipt of primary care | 2013 | DiD |  |
| Sadhu, A. R.; Ang, A. C.; Ingram-Drake, L. A.; Martinez, D. S.; Hsueh, W. A.; Ettner, S. L. | Economic benefits of intensive insulin therapy in critically Ill patients: The targeted insulin therapy to improve hospital outcomes (TRIUMPH) project | 2008 | DiD |  |
| Sarkar, U.; Lyles, C. R.; Parker, M. M.; Allen, J.; Nguyen, R.; Moffet, H. H.; Schillinger, D.; Karter, A. J. | Use of the refill function through an online patient portal is associated with improved adherence to statins in an integrated health system | 2014 | DiD |  |
| Watt, C.; Abuya, T.; Warren, C. E.; Obare, F.; Kanya, L.; Bellows, B. | Can reproductive health voucher programs improve quality of postnatal care? A quasi-experimental evaluation of Kenya ' s Safe Motherhood voucher scheme | 2015 | DiD |  |
| De Preux, L. B. | Anticipatory ex ante moral hazard and the effect of medicare on prevention | 2011 | DiD; DiDiD |  |
| Rajaram, R.; Chung, J. W.; Jones, A. T.; Cohen, M. E.; Dahlke, A. R.; Ko, C. Y.; Tarpley, J. L.; Lewis, F. R.; Hoyt, D. B.; Bilimoria, K. Y. | Association of the 2011 ACGME resident duty hour reform with general surgery patient outcomes and with resident examination performance | 2014 | DiD; DiDiD |  |
| Domino, M. E.; Norton, E. C.; Morrissey, J. P.; Thakur, N. | Cost shifting to jails after a change to managed mental health care | 2004 | DiD; Fixed effects |  |
| Hodgkin, D.; Parks Thomas, C.; Simoni-Wastila, L.; Ritter, G. A.; Lee, S. | The effect of a three-tier formulary on antidepressant utilization and expenditures | 2008 | Fixed effects |  |
| Li, J.; Hurley, J.; DeCicca, P.; Buckley, G. | Physician response to pay-for-performance: evidence from a natural experiment | 2014 | DiD pooled OLS; DiD (Fixed effects); DiD + differential trends |  |
| Yoon, J.; Bernell, S. L. | The role of adverse physical health events on the utilization of mental health services | 2013 | DiD & Fixed Effects |  |
| Fortney, J. C.; Steffick, D. E.; Burgess Jr, J. F.; Maciejewski, M. L.; Petersen, L. A. | Are primary care services a substitute or complement for specialty and inpatient services? | 2005 | IVA applied to DiD | Geographic |
| Hay, J.; Jhaveri, M.; Tangirala, M.; Kaliner, M. | Cost and resource utilization comparisons of second-generation antihistamines vs. montelukast for allergic rhinitis treatment | 2009 | IVA applied to Fixed effects | Historical |
| Chung, S.; Domino, M. E.; Stearns, S. C. | The effect of retirement on weight | 2009 | Fixed Effects; IVA applied to Fixed effects | Lagged |
| Wagner, T. H.; Jimison, H. B. | Computerized health information and the demand for medical care | 2003 | IVA applied to Fixed effects | Other |
| Kawatkar, A. A.; Hay, J. W.; Stohl, W.; Nichol, M. B. | Incremental expenditure of biologic disease modifying antirheumatic treatment using instrumental variables in panel data | 2013 | Dynamic panel model (IV-GMM) | Lagged |
| Piernas, C.; Ng, S. W.; Mendez, M. A.; Gordon-Larsen, P.; Popkin, B. M. | A dynamic panel model of the associations of sweetened beverage purchases with dietary quality and food-purchasing patterns | 2015 | Dynamic panel model (IV-GMM) | Lagged |
| O'Malley, A. J. | Instrumental variable specifications and assumptions for longitudinal analysis of mental health cost offsets | 2012 | IVA: IVA applied to Fixed effects | Lagged |
| Lei, X.; Lin, W. | The new cooperative medical scheme in rural China: Does more coverage mean more service and better health? | 2009 | Fixed effects; IVA; DiD | Geographic |
| Lin, M. J.; Liu, J. T. | Do lower birth weight babies have lower grades? Twin fixed effect and instrumental variable method evidence from Taiwan | 2009 | Fixed effects; IVA | Geographic |
| Schmittdiel, J. A.; Karter, A. J.; Dyer, W.; Parker, M.; Uratsu, C.; Chan, J.; Duru, O. K. | The comparative effectiveness of mail order pharmacy use vs. local pharmacy use on LDL-C control in new statin users | 2011 | DiD; IVA | Other |
| Basu, A. | Estimating Decision-Relevant Comparative Effects Using Instrumental Variables | 2011 | IVA | Geographic |
| Beck, C. A.; Penrod, J.; Gyorkos, T. W.; Shapiro, S.; Pilote, L. | Does Aggressive Care Following Acute Myocardial Infarction Reduce Mortality? Analysis with Instrumental Variables to Compare Effectiveness in Canadian and United States Patient Populations | 2003 | IVA | Geographic |
| Chen, L. F.; Chen, H. P.; Huang, Y. S.; Huang, K. Y.; Chou, P.; Lee, C. C. | Pneumococcal Pneumonia and the Risk of Stroke: A Population-Based Follow-Up Study | 2012 | IVA | Geographic |
| Edwards, S. T.; Prentice, J. C.; Simon, S. R.; Pizer, S. D. | Home-Based Primary Care and the risk of ambulatory care-sensitive condition hospitalization among older veterans with diabetes mellitus | 2014 | IVA | Geographic |
| Frances, C. D.; Shlipak, M. G.; Noguchi, H.; Heidenreich, P. A.; McClellan, M. | Does physician specialty affect the survival of elderly patients with myocardial infarction? | 2000 | IVA | Geographic |
| Goldman, D. P.; Bao, Y. | Effective HIV treatment and the employment of HIV+ adults | 2004 | IVA | Geographic |
| Gowrisankaran, G.; Town, R. J. | Estimating the quality of care in hospitals using instrumental variables | 1999 | IVA | Geographic |
| Hirth, R. A.; Grabowski, D. C.; Feng, Z.; Rahman, M.; Mor, V. | Effect of nursing home ownership on hospitalization of long-stay residents: An instrumental variables approach | 2014 | IVA | Geographic |
| Kahn, J. M.; Werner, R. M.; David, G.; Ten Have, T. R.; Benson, N. M.; Asch, D. A. | Effectiveness of long-term acute care hospitalization in elderly patients with chronic critical illness | 2013 | IVA | Geographic |
| Linden, A.; Adams, J. L. | Evaluating disease management programme effectiveness: An introduction to instrumental variables | 2006 | IVA | Geographic |
| Norton, E. C.; Lindrooth, R. C.; Ennett, S. T. | Controlling for the endogeneity of peer substance use on adolescent alcohol and tobacco use | 1998 | IVA | Geographic |
| Pilote, L.; Beck, C. A.; Eisenberg, M. J.; Humphries, K.; Joseph, L.; Penrod, J. R.; Tu, J. V. | Comparing invasive and noninvasive management strategies for acute myocardial infarction using administrative databases | 2008 | IVA | Geographic |
| Pracht, E. E.; Tepas, Iii J. J.; Celso, B. G.; Langland-Orban, B.; Flint, L. | Survival advantage associated with treatment of injury at designated trauma centers: A bivariate probit model with instrumental variables | 2007 | IVA | Geographic |
| Slade, E. P.; McCarthy, J. F.; Valenstein, M.; Visnic, S.; Dixon, L. B. | Cost savings from assertive community treatment services in an era of declining psychiatric inpatient use | 2013 | IVA | Geographic |
| Tsai, A. C.; Votruba, M.; Bridges, J. F. P.; Cebul, R. D. | Overcoming bias in estimating the volume-outcome relationship | 2006 | IVA | Geographic |
| Wehby, G. L.; Ullrich, F.; Xie, Y. | Very low birth weight hospital volume and mortality: An instrumental variables approach | 2012 | IVA | Geographic |
| Hadley, J.; Polsky, D.; Mandelblatt, J. S.; Mitchell, J. M.; Weeks, J. C.; Wang, Q.; Hwang, Y. T. | An exploratory instrumental variable analysis of the outcomes of localized breast cancer treatments in a medicare population | 2003 | IVA | Geographic + Historical + Time |
| O'Malley, A. J.; Frank, R. G.; Normand, S. L. T. | Estimating cost-offsets of new medications: Use of new antipsychotics and mental health costs for schizophrenia | 2011 | IVA | Geographic + Time |
| Abrahamowicz, M.; Beauchamp, M. E.; Ionescu-Ittu, R.; Delaney, J. A. C.; Pilote, L. | Reducing the variance of the prescribing preference-based instrumental variable estimates of the treatment effect | 2011 | IVA | Historical |
| An, J.; Nichol, M. B. | Multiple medication adherence and its effect on clinical outcomes among patients with comorbid type 2 diabetes and hypertension | 2013 | IVA | Historical |
| Bekelman, J. E.; Mitra, N.; Handorf, E. A.; Uzzo, R. G.; Hahn, S. A.; Polsky, D.; Armstrong, K. | Effectiveness of androgen-deprivation therapy and radiotherapy for older men with locally advanced prostate cancer | 2015 | IVA | Historical |
| Bhowmik, D.; Aparasu, R. R.; Rajan, S. S.; Sherer, J. T.; Ochoa-Perez, M.; Chen, H. | Risk of manic switch associated with antidepressant therapy in pediatric bipolar depression | 2014 | IVA | Historical |
| Brooks, J. M.; Tang, Y.; Chapman, C. G.; Cook, E. A.; Chrischilles, E. A. | What is the effect of area size when using local area practice style as an instrument? | 2013 | IVA | Historical |
| Chuang, C. M.; Chou, Y. J.; Yen, M. S.; Chao, K. C.; Twu, N. F.; Wu, H. H.; Wen, K. C.; Chen, Y. J.; Wang, P. H.; Lai, C. R.; Chou, P. | The role of secondary cytoreductive surgery in patients with recurrent epithelial ovarian, tubal, and peritoneal cancers: A comparative effectiveness analysis | 2012 | IVA | Historical |
| De Ridder, A.; De Graeve, D. | Can we account for selection bias? A comparison between bare metal and drug-eluting stents | 2011 | IVA | Historical |
| Fang, G.; Brooks, J. M.; Chrischilles, E. A. | Comparison of instrumental variable analysis using a new instrument with risk adjustment methods to reduce confounding by indication | 2012 | IVA | Historical |
| Figueroa, R.; Harman, J.; Engberg, J. | Use of Claims Data to Examine the Impact of Length of Inpatient Psychiatric Stay on Readmission Rate | 2004 | IVA | Historical |
| Huesch, M. D. | External adjustment sensitivity analysis for unmeasured confounding: An application to coronary stent outcomes, Pennsylvania 2004-2008 | 2013 | IVA | Historical |
| Huybrechts, K. F.; Brookhart, M. A.; Rothman, K. J.; Silliman, R. A.; Gerhard, T.; Crystal, S.; Schneeweiss, S. | Comparison of different approaches to confounding adjustment in a study on the association of antipsychotic medication with mortality in older nursing home patients | 2011 | IVA | Historical |
| Ionescu-Ittu, R. | Treatment effect estimates varied depending on the definition of the provider prescribing preference-based instrumental variables | 2012 | IVA | Historical |
| Kivimaki, M.; Vahtera, J.; Kawachi, I.; Ferrie, J. E.; Oksanen, T.; Joensuu, M.; Pentti, J.; Salo, P.; Elovainio, M.; Virtanen, M. | Psychosocial work environment as a risk factor for absence with a psychiatric diagnosis: An instrumental-variables analysis | 2010 | IVA | Historical |
| Kramer, A.; Jager, K. J.; Fogarty, D. G.; Ravani, P.; Finne, P.; Perez-Panades, J.; Prutz, K. G.; Arias, M.; Heaf, J. G.; Wanner, C.; Stel, V. S. | Association between pre-transplant dialysis modality and patient and graft survival after kidney transplantation | 2012 | IVA | Historical |
| Kuo, Y. F.; Montie, J. E.; Shahinian, V. B. | Reducing bias in the assessment of treatment effectiveness: Androgen deprivation therapy for prostate cancer | 2012 | IVA | Historical |
| Lakdawalla, D. N.; Mascarenhas, M.; Jena, A. B.; Vanderpuye-Orgle, J.; Lavallee, C.; Linthicum, M. T.; Snider, J. T. | Impact of oral nutrition supplements on hospital outcomes in pediatric patients | 2014 | IVA | Historical |
| MacKenzie, T. A.; Tosteson, T. D.; Morden, N. E.; Stukel, T. A.; O'Malley, A. J. | Using instrumental variables to estimate a Cox's proportional hazards regression subject to additive confounding | 2014 | IVA | Historical |
| Margolis, D. J.; Gupta, J.; Hoffstad, O.; Papdopoulos, M.; Glick, H. A.; Thom, S. R.; Mitra, N. | Lack of effectiveness of hyperbaric oxygen therapy for the treatment of diabetic foot ulcer and the prevention of amputation a cohort study | 2013 | IVA | Historical |
| Parmar, A. D.; Sheffield, K. M.; Han, Y.; Vargas, G. M.; Guturu, P.; Kuo, Y. F.; Goodwin, J. S.; Riall, T. S. | Evaluating comparative effectiveness with observational data: Endoscopic ultrasound and survival in pancreatic cancer | 2013 | IVA | Historical |
| Pisoni, R. L.; Arrington, C. J.; Albert, J. M.; Ethier, J.; Kimata, N.; Krishnan, M.; Rayner, H. C.; Saito, A.; Sands, J. J.; Saran, R.; Gillespie, B.; Wolfe, R. A.; Port, F. K. | Facility Hemodialysis Vascular Access Use and Mortality in Countries Participating in DOPPS: An Instrumental Variable Analysis | 2009 | IVA | Historical |
| Prentice, J. C.; Conlin, P. R.; Gellad, W. F.; Edelman, D.; Lee, T. A.; Pizer, S. D. | Capitalizing on prescribing pattern variation to compare medications for type 2 diabetes | 2014 | IVA | Historical |
| Rassen, J. A.; Brookhart, M. A.; Glynn, R. J.; Mittleman, M. A.; Schneeweiss, S. | Instrumental variables II: instrumental variable application-in 25 variations, the physician prescribing preference generally was strong and reduced covariate imbalance | 2009 | IVA | Historical |
| Rosenthal, M. B.; Li, Z.; Robertson, A. D.; Milstein, A. | Impact of financial incentives for prenatal care on birth outcomes and spending | 2009 | IVA | Historical |
| Sheffield, K. M.; Riall, T. S.; Han, Y.; Kuo, Y. F.; Townsend, C. M., Jr.; Goodwin, J. S. | Association between cholecystectomy with vs without intraoperative cholangiography and risk of common duct injury | 2013 | IVA | Historical |
| Steingrub, J. S.; Lagu, T.; Rothberg, M. B.; Nathanson, B. H.; Raghunathan, K.; Lindenauer, P. K. | Treatment with neuromuscular blocking agents and the risk of in-hospital mortality among mechanically ventilated patients with severe sepsis | 2014 | IVA | Historical |
| Stukel, Thérèse A; Fisher, Elliott S; Wennberg, David E; Alter, David A; Gottlieb, Daniel J; Vermeulen, Marian J | Analysis of observational studies in the presence of treatment selection bias: effects of invasive cardiac management on AMI survival using propensity score and instrumental variable methods. | 2007 | IVA | Historical |
| Tagami, T.; Matsui, H.; Horiguchi, H.; Fushimi, K.; Yasunaga, H. | Antithrombin and mortality in severe pneumonia patients with sepsis-associated disseminated intravascular coagulation: An observational nationwide study | 2014 | IVA | Historical |
| VanDyke, R. D.; McPhail, G. L.; Huang, B.; Fenchel, M. C.; Amin, R. S.; Carle, A. C.; Chini, B. A.; Seid, M. | Inhaled tobramycin effectively reduces FEV1 decline in cystic fibrosis an instrumental variables analysis | 2013 | IVA | Historical |
| Wong, K.; Campitelli, M. A.; Stukel, T. A.; Kwong, J. C. | Estimating influenza vaccine effectiveness in community-dwelling elderly patients using the instrumental variable analysis method | 2012 | IVA | Historical |
| Chen, H.; Mehta, S.; Aparasu, R.; Patel, A.; Ochoa-Perez, M. | Comparative effectiveness of monotherapy with mood stabilizers versus second generation (atypical) antipsychotics for the treatment of bipolar disorder in children and adolescents | 2014 | IVA | Historical + Time |
| Newman, T. B.; Vittinghoff, E.; McCulloch, C. E. | Efficacy of phototherapy for newborns with hyperbilirubinemia: a cautionary example of an instrumental variable analysis | 2012 | IVA | Historical + Time |
| Ahern, T. P.; Pedersen, L.; Svaerke, C.; Rothman, K. J.; Sorensen, H. T.; Lash, T. L. | The association between vitamin K antagonist therapy and site-specific cancer incidence estimated by using heart valve replacement as an instrumental variable | 2011 | IVA | Lagged |
| Cai, B.; Hennessy, S.; Flory, J. H.; Sha, D.;Ten Have, T. R.; Small, D. S. | Simulation study of instrumental variable approaches with an application to a study of the antidiabetic effect of bezafibrate | 2012 | IVA | Lagged |
| Cawley, J.; Meyerhoefer, C. | The medical care costs of obesity: An instrumental variables approach | 2012 | IVA | Other |
| Groenwold, R. H.; Hak, E.; Klungel, O. H.; Hoes, A. W. | Instrumental variables in influenza vaccination studies: mission impossible?! | 2010 | IVA | Other |
| Kim, D.; Leigh, J. P. | Estimating the effects of wages on obesity | 2010 | IVA | Other |
| Pirracchio, R.; Sprung, C.; Payen, D.; Chevret, S. | Benefits of ICU admission in critically ill patients: whether instrumental variable methods or propensity scores should be used | 2011 | IVA | Other |
| Selden, T. M.; Hudson, J. L. | Access to care and utilization among children: Estimating the effects of public and private coverage | 2006 | IVA | Other |
| Slade, E. P.; Wissow, L. S.; Davis, M.; Abrams, M. T.; Dixon, L. B. | Medicaid lapses and low-income young adults' receipt of outpatient mental health care after an inpatient stay | 2014 | IVA | Other |
| Hay, J. W.; Lawler, E.; Yucel, K.; Guo, A.; Balzer, T.; Gaziano, J. M.; Scranton, R. E. | Cost impact of diagnostic imaging for lower extremity peripheral vascular occlusive disease | 2009 | IVA | PScore (historical EHRs) |
| Guo, J.; Konetzka, R. T.; Manning, W. G. | The causal effects of home care use on institutional long-term care utilization and expenditures | 2015 | IVA | Randomisation |
| Federspiel, J. J.; Stearns, S. C.; Sheridan, B. C.; Kuritzky, J. J.; D'Arcy, L. P.; Crespin, D. J.; Carey, T. S.; Rossi, J. S. | Evaluating the effectiveness of a rapidly adopted cardiovascular technology with administrative data: The case of drug-eluting stents for acute coronary syndromes | 2012 | IVA | Time |
| Goyal, N.; Zubizarreta, J. R.; Small, D. S.; Lorch, S. A. | Length of stay and readmission among late preterm infants: An instrumental variable approach | 2013 | IVA | Time |
| Hollingsworth, J. M.; Norton, E. C.; Kaufman, S. R.; Smith, R. M.; Wolf Jr, J. S.; Hollenbeck, B. K. | Medical expulsive therapy versus early endoscopic stone removal for acute renal colic: An instrumental variable analysis | 2013 | IVA | Time |
| Johnston, K. M.; Gustafson, P.; Levy, A. R.; Grootendorst, P. | Use of instrumental variables in the analysis of generalized linear models in the presence of unmeasured confounding with applications to epidemiological research | 2008 | IVA | Time |
| O'Donnell, H. C.; Colman, G.; Trachtman, R. A.; Velazco, N.; Racine, A. D. | Impact of newborn follow-up visit timing on subsequent ED visits and hospital readmissions: AN instrumental variable analysis | 2014 | IVA | Time |
| Zeliadt, S. B.; Loggers, E. T.; Slatore, C. G.; Au, D. H.; Hebert, P. L.; Klein, G. J.; Kessler, L. G.; Backhus, L. M. | Preoperative PET and the reduction of unnecessary surgery among newly diagnosed lung cancer patients in a community setting | 2014 | IVA | Time |
| Brunner, E. J.; Kivimaki, M.; Witte, D. R.; Lawlor, D. A.; Davey Smith, G.; Cooper, J. A.; Miller, M.; Lowe, G. D.; Rumley, A.; Casas, J. P.; Shah, T.; Humphries, S. E.; Hingorani, A. D.; Marmot, M. G.; Timpson, N. J.; Kumari, M. | Inflammation, insulin resistance, and diabetes--Mendelian randomization using CRP haplotypes points upstream | 2008 | IVA (Mendelian) | Mendelian |
| Burgess, S.; Thompson, S. G. | Avoiding bias from weak instruments in mendelian randomization studies | 2011 | IVA (Mendelian) | Mendelian |
| Haring, R.; Teumer, A.; Volker, U.; Dorr, M.; Nauck, M.; Biffar, R.; Volzke, H.; Baumeister, S. E.; Wallaschofski, H. | Mendelian randomization suggests non-causal associations of testosterone with cardiometabolic risk factors and mortality | 2013 | IVA (Mendelian) | Mendelian |
| Jokela, M.; Elovainio, M.; Keltikangas-Jarvinen, L.; Batty, G. D.; Hintsanen, M.; Seppala, I.; Kahonen, M.; Viikari, J. S.; Raitakari, O. T.; Lehtimaki, T.; Kivimaki, M. | Body mass index and depressive symptoms: Instrumental-variables regression with genetic risk score | 2012 | IVA (Mendelian) | Mendelian |
| Kivimaki, M.; Magnussen, C. G.; Juonala, M.; Kahonen, M.; Kettunen, J.; Loo, B. M.; Lehtimaki, T.; Viikari, J.; Raitakari, O. T. | Conventional and Mendelian randomization analyses suggest no association between lipoprotein(a) and early atherosclerosis: The Young Finns Study | 2011 | IVA (Mendelian) | Mendelian |
| Laschkolnig, A.; Kollerits, B.; Lamina, C.; Meisinger, C.; Rantner, B.; Stadler, M.; Peters, A.; Koenig, W.; Stockl, A.; Dahnhardt, D.; Boger, C. A.; Kramer, B. K.; Fraedrich, G.; Strauch, K.; Kronenberg, F. | Lipoprotein (a) concentrations, apolipoprotein (a) phenotypes, and peripheral arterial disease in three independent cohorts | 2014 | IVA (Mendelian) | Mendelian |
| Lawlor, D. A.; Harbord, R. M.; Timpson, N. J.; Lowe, G. D.; Rumley, A.; Gaunt, T. R.; Baker, I.; Yarnell, J. W.; Kivimaki, M.; Kumari, M.; Norman, P. E.; Jamrozik, K.; Hankey, G. J.; Almeida, O. P.; Flicker, L.; Warrington, N.; Marmot, M. G.; Ben-Shlomo, Y.; Palmer, L. J.; Day, I. N.; Ebrahim, S.; Smith, G. D. | The association of C-reactive protein and CRP genotype with coronary heart disease: findings from five studies with 4,610 cases amongst 18,637 participants | 2008 | IVA (Mendelian) | Mendelian |
| Leong, A.; Rehman, W.; Dastani, Z.; Greenwood, C.; Timpson, N.; Langsetmo, L.; Berger, C.; Fu, L.; Wong, B. Y. L.; Malik, S.; Malik, R.; Hanley, D. A.; Cole, D. E. C.; Goltzman, D.; Richards, J. B. | The Causal Effect of Vitamin D Binding Protein (DBP) Levels on Calcemic and Cardiometabolic Diseases: A Mendelian Randomization Study | 2014 | IVA (Mendelian) | Mendelian |
| Nimptsch, K.; Aleksandrova, K.; Boeing, H.; Janke, J.; Lee, Y. A.; Jenab, M.; Bueno-De-Mesquita, H. B.; Jansen, E. H. J. M.; Tsilidis, K. K.; Trichopoulou, A.; Weiderpass, E.; Wu, C.; Overvad, K.; Tjonneland, A.; Boutron-Ruault, M. C.; Dossus, L.; Racine, A.; Kaaks, R.; Canzian, F.; Lagiou, P.; Trichopoulos, D.; Palli, D.; Agnoli, C.; Tumino, R.; Vineis, P.; Panico, S.; Johansson, A.; Van Guelpen, B.; Khaw, K. T.; Wareham, N.; Peeters, P. H.; Quiros, J. R.; Garcia, A. V.; Molina-Montes, E.; Dorronsoro, M.; Chirlaque, M. D.; Gurrea, A. B.; Key, T. J.; Duarte-Salles, T.; Stepien, M.; Gunter, M. J.; Riboli, E.; Pischon, T. | Association of CRP genetic variants with blood concentrations of C-reactive protein and colorectal cancer risk | 2015 | IVA (Mendelian) | Mendelian |
| Palmer, T. M.; Sterne, J. A. C.; Harbord, R. M.; Lawlor, D. A.; Sheehan, N. A.; Meng, S.; Granell, R.; Smith, G. D.; Didelez, V. | Instrumental variable estimation of causal risk ratios and causal odds ratios in mendelian randomization analyses | 2011 | IVA (Mendelian) | Mendelian |
| Wehby, G. L.; Scholder, Sv | Genetic instrumental variable studies of effects of prenatal risk factors | 2013 | IVA (Mendelian) | Mendelian |
| Richardson, D. B.; Laurier, D.; Schubauer-Berigan, M. K.; Tchetgen, E. T.; Cole, S. R. | Assessment and indirect adjustment for confounding by smoking in cohort studies using relative hazards models | 2014 | Negative Control Outcome |  |
| Brophy, S.; Jones, K. H.; Rahman, M. A.; Zhou, S. M.; John, A.; Atkinson, M. D.; Francis, N.; Lyons, R. A.; Dunstan, F. | Incidence of campylobacter and salmonella infections following first prescription for PPI: A cohort study using routine data | 2013 | PERR |  |
| Tannen, R. L. | Use of primary care electronic medical record database in drug efficacy research on cardiovascular outcomes: Comparison of database and randomised controlled trial findings | 2009 | PERR |  |
| Tannen, R. L.; Weiner, M. G.; Xie, D. | Replicated studies of two randomized trials of angiotensin-converting enzyme inhibitors: Further empiric validation of the 'prior event rate ratio' to adjust for unmeasured confounding by indication | 2008 | PERR |  |
| Tannen, R.; Xie, D.; Wang, X.; Yu, M.; Weiner, M. G. | A new "Comparative Effectiveness" assessment strategy using the THIN database: Comparison of the cardiac complications of pioglitazone and rosiglitazone | 2013 | PERR |  |
| Uddin, M. J.; Groenwold, R. H. H.; Van Staa, T. P.; De Boer, A.; Belitser, S. V.; Hoes, A. W.; Roes, K. C. B.; Klungel, O. H. | Performance of prior event rate ratio adjustment method in pharmacoepidemiology: A simulation study | 2015 | PERR |  |
| Lee, W. C. | Detecting and correcting the bias of unmeasured factors using perturbation analysis: a data-mining approach | 2014 | Perturbation analysis |  |
| Lunt, M.; Glynn, R. J.; Rothman, K. J.; Avorn, J.; Sturmer, T. | Propensity score calibration in the absence of surrogacy | 2012 | PSC |  |
| Sturmer, T. | Performance of propensity score calibration - A simulation study | 2007 | PSC |  |
| Stürmer, Til, Schneeweiss, Sebastian, Avorn, Jerry; | Adjusting effect estimates for unmeasured confounding with validation data using propensity score calibration | 2005 | PSC |  |
| Albouy, V.; Lequien, L. | Does compulsory education lower mortality? | 2009 | RD |  |
| Swaminathan, S.; Mor, V.; Mehrotra, R.; Trivedi, A. N. | Effect of medicare dialysis payment reform on use of erythropoiesis stimulating agents | 2015 | RD |  |
| Zuckerman, I. H.; Lee, E.; Wutoh, A. K.; Xue, Z.; Stuart, B. | Application of regression-discontinuity analysis in pharmaceutical health services research | 2006 | RD |  |
