# Supplementary material for: Adjusting for unmeasured confounding in nonrandomized longitudinal studies: a methodological review
Source: J Clin Epidemiol. 2017 Jul;87:23–34. doi: 10.1016/j.jclinepi.2017.04.022 (PMC5589113; doi:10.1016/j.jclinepi.2017.04.022)
Supplement: Supplementary References [file mmc3.docx]

# References (included studies)

1. Stürmer T, Schneeweiss S, Avorn J, Glynn RJ. Adjusting effect estimates for unmeasured confounding with validation data using propensity score calibration. *Am J Epidemiol*. 2005;162(3):279-289. doi:10.1093/aje/kwi192.
2. Stürmer T, Schneeweiss S, Rothman KJ, Avorn J, Glynn RJ. Performance of propensity score calibration - A simulation study. *Am J Epidemiol*. 2007;165(10):1110-1118. doi:http://dx.doi.org/10.1093/aje/kwm074.
3. Lunt M, Glynn RJ, Rothman KJ, Avorn J, Stürmer T. Propensity score calibration in the absence of surrogacy. *Am J Epidemiol*. 2012;175(12):1294-1302. doi:10.1093/aje/kwr463.
4. Lee W-C. Detecting and correcting the bias of unmeasured factors using perturbation analysis: a data-mining approach. *BMC Med Res Methodol*. 2014;14(1):18. doi:10.1186/1471-2288-14-18.
5. Brunner EJ, Kivimaki M, Witte DR, et al. Inflammation, insulin resistance, and diabetes--Mendelian randomization using CRP haplotypes points upstream. *PLoS Med*. 2008;5(8):e155. internal-pdf://137.108.247.40/Infl_diabet_MendRand-Brunner-PLoS08.pdf.
6. Burgess S, Thompson SG. Avoiding bias from weak instruments in mendelian randomization studies. *Int J Epidemiol*. 2011;40(3):755-764. doi:http://dx.doi.org/10.1093/ije/dyr036.
7. Haring R, Teumer A, Völker U, et al. Mendelian randomization suggests non-causal associations of testosterone with cardiometabolic risk factors and mortality. *Andrology*. 2013;1(1):17-23. doi:10.1111/j.2047-2927.2012.00002.x.
8. Jokela M, Elovainio M, Keltikangas-Jarvinen L, et al. Body mass index and depressive symptoms: Instrumental-variables regression with genetic risk score. *Genes, Brain Behav*. 2012;11(8):942-948. doi:http://dx.doi.org/10.1111/j.1601-183X.2012.00846.x.
9. Kivimaki M, Magnussen CG, Juonala M, et al. Conventional and Mendelian randomization analyses suggest no association between lipoprotein(a) and early atherosclerosis: The Young Finns Study. *Int J Epidemiol*. 2011;40(2):470-478. doi:http://dx.doi.org/10.1093/ije/dyq205.
10. Laschkolnig A, Kollerits B, Lamina C, et al. Lipoprotein (a) concentrations, apolipoprotein (a) phenotypes, and peripheral arterial disease in three independent cohorts. *Cardiovasc Res*. 2014;103(1):28-36. doi:http://dx.doi.org/10.1093/cvr/cvu107.
11. Lawlor DA, Harbord RM, Timpson NJ, et al. The association of C-reactive protein and CRP genotype with coronary heart disease: findings from five studies with 4,610 cases amongst 18,637 participants. *PLoS ONE [Electronic Resour*. 2008;3(8):e3011. doi:http://dx.doi.org/10.1371/journal.pone.0003011.
12. Leong A, Rehman W, Dastani Z, et al. The Causal Effect of Vitamin D Binding Protein (DBP) Levels on Calcemic and Cardiometabolic Diseases: A Mendelian Randomization Study. *PLoS Med*. 2014;11(10):e1001751. doi:http://dx.doi.org/10.1371/journal.pmed.1001751.
13. Nimptsch K, Aleksandrova K, Boeing H, et al. Association of CRP genetic variants with blood concentrations of C-reactive protein and colorectal cancer risk. *Int J Cancer*. 2015;136(5):1181-1192. doi:http://dx.doi.org/10.1002/ijc.29086.
14. Palmer TM, Sterne JAC, Harbord RM, et al. Instrumental variable estimation of causal risk ratios and causal odds ratios in mendelian randomization analyses. *Am J Epidemiol*. 2011;173(12):1392-1403. doi:http://dx.doi.org/10.1093/aje/kwr026.
15. Wehby GL, Scholder S. Genetic instrumental variable studies of effects of prenatal risk factors. *Biodemography Soc Biol*. 2013;59(1):4-36. doi:http://dx.doi.org/10.1080/19485565.2013.774615.
16. Basu A. Estimating Decision-Relevant Comparative Effects Using Instrumental Variables. *Stat Biosci*. 2011;3(1):6-27. doi:http://dx.doi.org/10.1007/s12561-011-9033-6.
17. Beck CA, Penrod J, Gyorkos TW, Shapiro S, Pilote L. Does Aggressive Care Following Acute Myocardial Infarction Reduce Mortality? Analysis with Instrumental Variables to Compare Effectiveness in Canadian and United States Patient Populations. *Health Serv Res*. 2003;38(6):1423-1440. internal-pdf://133.21.114.118/MI_mort_IV-Beck-HSR03.pdf.
18. Cawley J, Meyerhoefer C. The medical care costs of obesity: An instrumental variables approach. *J Health Econ*. 2012;31:219-230. doi:http://dx.doi.org/10.1016/j.jhealeco.2011.10.003.
19. Chen LF, Chen HP, Huang YS, Huang KY, Chou P, Lee CC. Pneumococcal Pneumonia and the Risk of Stroke: A Population-Based Follow-Up Study. *PLoS One*. 2012;7(12):e51452. doi:http://dx.doi.org/10.1371/journal.pone.0051452.
20. Edwards ST, Prentice JC, Simon SR, Pizer SD. Home-Based Primary Care and the risk of ambulatory care-sensitive condition hospitalization among older veterans with diabetes mellitus. *JAMA Intern Med*. 2014;174(11):1796-1803. doi:http://dx.doi.org/10.1001/jamainternmed.2014.4327.
21. Federspiel JJ, Stearns SC, Sheridan BC, et al. Evaluating the effectiveness of a rapidly adopted cardiovascular technology with administrative data: The case of drug-eluting stents for acute coronary syndromes. *Am Heart J*. 2012;164(2):207-214. doi:http://dx.doi.org/10.1016/j.ahj.2012.05.016.
22. Frances CD, Shlipak MG, Noguchi H, Heidenreich PA, McClellan M. Does physician specialty affect the survival of elderly patients with myocardial infarction? *Health Serv Res*. 2000;35(5):1093-1116. http://ovidsp.ovid.com/ovidweb.cgi?T=JS&CSC=Y&NEWS=N&PAGE=fulltext&D=emed5&AN=2001006754.
23. Goldman DP, Bao Y. Effective HIV treatment and the employment of HIV+ adults. *Health Serv Res*. 2004;39(6):1691-1712. http://ovidsp.ovid.com/ovidweb.cgi?T=JS&CSC=Y&NEWS=N&PAGE=fulltext&D=emed6&AN=2004522727; http://lib.exeter.ac.uk:4556/resserv?sid=OVID:embase&id=pmid:&id=doi:&issn=0017-9124&isbn=&volume=39&issue=6+I&spage=1691&pages=1691-1712&date=2004&title=Health+Serv.
24. Gowrisankaran G, Town RJ. Estimating the quality of care in hospitals using instrumental variables. *J Health Econ*. 1999;18(6):747-767. doi:http://dx.doi.org/10.1016/S0167-6296%2899%2900022-3.
25. Goyal N, Zubizarreta JR, Small DS, Lorch SA. Length of stay and readmission among late preterm infants: An instrumental variable approach. *Hosp Pediatr*. 2013;3(1):7-15. doi:http://dx.doi.org/10.1542/hpeds.2012-0027.
26. Groenwold RH, Hak E, Klungel OH, Hoes AW. Instrumental variables in influenza vaccination studies: mission impossible?! *Value Heal*. 2010;13(1):132-137. doi:http://dx.doi.org/10.1111/j.1524-4733.2009.00584.x.
27. Hirth RA, Grabowski DC, Feng Z, Rahman M, Mor V. Effect of nursing home ownership on hospitalization of long-stay residents: An instrumental variables approach. *Int J Health Care Finance Econ*. 2014;14(1):1-18. doi:http://dx.doi.org/10.1007/s10754-013-9136-3.
28. Hollingsworth JM, Norton EC, Kaufman SR, Smith RM, Wolf Jr JS, Hollenbeck BK. Medical expulsive therapy versus early endoscopic stone removal for acute renal colic: An instrumental variable analysis. *J Urol*. 2013;190(3):882-887. doi:http://dx.doi.org/10.1016/j.juro.2013.03.040.
29. Johnston KM, Gustafson P, Levy AR, Grootendorst P. Use of instrumental variables in the analysis of generalized linear models in the presence of unmeasured confounding with applications to epidemiological research. *Stat Med*. 2008;27(9):1539-1556. doi:10.1002/sim.3036.
30. Kahn JM, Werner RM, David G, Ten Have TR, Benson NM, Asch DA. Effectiveness of long-term acute care hospitalization in elderly patients with chronic critical illness. *Med Care*. 2013;51(1):4-10. doi:http://dx.doi.org/10.1097/MLR.0b013e31826528a7.
31. Kim D, Leigh JP. Estimating the effects of wages on obesity. *J Occup Environ Med*. 2010;52(5):495-500. doi:http://dx.doi.org/10.1097/JOM.0b013e3181dbc867.
32. Lei X, Lin W. The new cooperative medical scheme in rural China: Does more coverage mean more service and better health? *Health Econ*. 2009;18(suppl. 2):S25-S46. doi:http://dx.doi.org/10.1002/hec.1501.
33. Lin MJ, Liu JT. Do lower birth weight babies have lower grades? Twin fixed effect and instrumental variable method evidence from Taiwan. *Soc Sci Med*. 2009;68(10):1780-1787. doi:http://dx.doi.org/10.1016/j.socscimed.2009.02.031.
34. Linden A, Adams JL. Evaluating disease management programme effectiveness: An introduction to instrumental variables. *J Eval Clin Pract*. 2006;12(2):148-154. doi:http://dx.doi.org/10.1111/j.1365-2753.2006.00615.x.
35. Norton EC, Lindrooth RC, Ennett ST. Controlling for the endogeneity of peer substance use on adolescent alcohol and tobacco use. *Health Econ*. 1998;7(5):439-453. doi:http://dx.doi.org/10.1002/%28SICI%291099-1050%28199808%297:5%3C439::AID-HEC362%3E3.0.CO;2-9.
36. O’Donnell HC, Colman G, Trachtman RA, Velazco N, Racine AD. Impact of newborn follow-up visit timing on subsequent ED visits and hospital readmissions: AN instrumental variable analysis. *Acad Pediatr*. 2014;14(1):84-91. doi:http://dx.doi.org/10.1016/j.acap.2013.09.010.
37. O’Malley AJ, Frank RG, Normand SLT. Estimating cost-offsets of new medications: Use of new antipsychotics and mental health costs for schizophrenia. *Stat Med*. 2011;30(16):1971-1988. doi:http://dx.doi.org/10.1002/sim.4245.
38. Pilote L, Beck CA, Eisenberg MJ, et al. Comparing invasive and noninvasive management strategies for acute myocardial infarction using administrative databases. *Am Heart J*. 2008;155(1):42-48. doi:http://dx.doi.org/10.1016/j.ahj.2007.09.016.
39. Pirracchio R, Sprung C, Payen D, Chevret S. Benefits of ICU admission in critically ill patients: whether instrumental variable methods or propensity scores should be used. *BMC Med Res Methodol*. 2011;11(132). http://ovidsp.ovid.com/ovidweb.cgi?T=JS&CSC=Y&NEWS=N&PAGE=fulltext&D=emed11&AN=21936926.
40. Pracht EE, Tepas IJJ, Celso BG, Langland-Orban B, Flint L. Survival advantage associated with treatment of injury at designated trauma centers: A bivariate probit model with instrumental variables. *Med Care Res Rev*. 2007;64(1):83-97. doi:http://dx.doi.org/10.1177/1077558706296241.
41. Schmittdiel JA, Karter AJ, Dyer W, et al. The comparative effectiveness of mail order pharmacy use vs. local pharmacy use on LDL-C control in new statin users. *J Gen Intern Med*. 2011;26(12):1396-1402. doi:http://dx.doi.org/10.1007/s11606-011-1805-7.
42. Selden TM, Hudson JL. Access to care and utilization among children: Estimating the effects of public and private coverage. *Med Care*. 2006;44(5 SUPPL.):I19-I26. doi:http://dx.doi.org/10.1097/01.mlr.0000208137.46917.3b.
43. Slade EP, McCarthy JF, Valenstein M, Visnic S, Dixon LB. Cost savings from assertive community treatment services in an era of declining psychiatric inpatient use. *Health Serv Res*. 2013;48(1):195-217. doi:http://dx.doi.org/10.1111/j.1475-6773.2012.01420.x.
44. Slade EP, Wissow LS, Davis M, Abrams MT, Dixon LB. Medicaid lapses and low-income young adults’ receipt of outpatient mental health care after an inpatient stay. *Psychiatr Serv*. 2014;65(4):454-460. doi:http://dx.doi.org/10.1176/appi.ps.201200375.
45. Tsai AC, Votruba M, Bridges JFP, Cebul RD. Overcoming bias in estimating the volume-outcome relationship. *Health Serv Res*. 2006;41(1):252-264. doi:http://dx.doi.org/10.1111/j.1475-6773.2005.00461.x.
46. Wehby GL, Ullrich F, Xie Y. Very low birth weight hospital volume and mortality: An instrumental variables approach. *Med Care*. 2012;50(8):714-721. doi:http://dx.doi.org/10.1097/MLR.0b013e31824e32cf.
47. Zeliadt SB, Loggers ET, Slatore CG, et al. Preoperative PET and the reduction of unnecessary surgery among newly diagnosed lung cancer patients in a community setting. *J Nucl Med*. 2014;55(3):379-385. doi:http://dx.doi.org/10.2967/jnumed.113.124230.
48. O’Malley AJ. Instrumental variable specifications and assumptions for longitudinal analysis of mental health cost offsets. *Heal Serv Outcomes Res Methodol*. 2012;12(4):254-272. doi:http://dx.doi.org/10.1007/s10742-012-0097-7.
49. Richardson DB, Laurier D, Schubauer-Berigan MK, Tchetgen ET, Cole SR. Assessment and indirect adjustment for confounding by smoking in cohort studies using relative hazards models. *Am J Epidemiol*. 2014;180(9):933-940. doi:http://dx.doi.org/10.1093/aje/kwu211.
50. Abrahamowicz M, Beauchamp ME, Ionescu-Ittu R, Delaney JAC, Pilote L. Reducing the variance of the prescribing preference-based instrumental variable estimates of the treatment effect. *Am J Epidemiol*. 2011;174(4):494-502. doi:http://dx.doi.org/10.1093/aje/kwr057.
51. Ahern TP, Pedersen L, Svaerke C, Rothman KJ, Sorensen HT, Lash TL. The association between vitamin K antagonist therapy and site-specific cancer incidence estimated by using heart valve replacement as an instrumental variable. *Am J Epidemiol*. 2011;174(12):1382-1390. doi:http://dx.doi.org/10.1093/aje/kwr268.
52. An J, Nichol MB. Multiple medication adherence and its effect on clinical outcomes among patients with comorbid type 2 diabetes and hypertension. *Med Care*. 2013;51(10):879-887. doi:http://dx.doi.org/10.1097/MLR.0b013e31829fa8ed.
53. Bekelman JE, Mitra N, Handorf EA, et al. Effectiveness of androgen-deprivation therapy and radiotherapy for older men with locally advanced prostate cancer. *J Clin Oncol*. 2015;33(7):716-722. doi:http://dx.doi.org/10.1200/JCO.2014.57.2743.
54. Bhowmik D, Aparasu RR, Rajan SS, Sherer JT, Ochoa-Perez M, Chen H. Risk of manic switch associated with antidepressant therapy in pediatric bipolar depression. *J Child Adolesc Psychopharmacol*. 2014;24(10):551-561. doi:http://dx.doi.org/10.1089/cap.2014.0028.
55. Brooks JM, Tang Y, Chapman CG, Cook EA, Chrischilles EA. What is the effect of area size when using local area practice style as an instrument? *J Clin Epidemiol*. 2013;66(8 SUPPL.8):S69-S83. doi:http://dx.doi.org/10.1016/j.jclinepi.2013.04.008.
56. Cai B, Hennessy S, Flory JH, Sha D, Ten Have TR, Small DS. Simulation study of instrumental variable approaches with an application to a study of the antidiabetic effect of bezafibrate. *Pharmacoepidemiol Drug Saf*. 2012;21(SUPPL.2):114-120. doi:http://dx.doi.org/10.1002/pds.3252.
57. Chen H, Mehta S, Aparasu R, Patel A, Ochoa-Perez M. Comparative effectiveness of monotherapy with mood stabilizers versus second generation (atypical) antipsychotics for the treatment of bipolar disorder in children and adolescents. *Pharmacoepidemiol Drug Saf*. 2014;23(3):299-308. doi:http://dx.doi.org/10.1002/pds.3568.
58. Chuang CM, Chou YJ, Yen MS, et al. The role of secondary cytoreductive surgery in patients with recurrent epithelial ovarian, tubal, and peritoneal cancers: A comparative effectiveness analysis. *Oncologist*. 2012;17(6):847-855. doi:http://dx.doi.org/10.1634/theoncologist.2011-0373.
59. De Ridder A, De Graeve D. Can we account for selection bias? A comparison between bare metal and drug-eluting stents. *Value Heal*. 2011;14(1):3-14. doi:http://dx.doi.org/10.1016/j.jval.2010.10.014.
60. Fang G, Brooks JM, Chrischilles EA. Comparison of instrumental variable analysis using a new instrument with risk adjustment methods to reduce confounding by indication. *Am J Epidemiol*. 2012;175(11):1142-1151. doi:http://dx.doi.org/10.1093/aje/kwr448.
61. Figueroa R, Harman J, Engberg J. Use of Claims Data to Examine the Impact of Length of Inpatient Psychiatric Stay on Readmission Rate. *Psychiatr Serv*. 2004;55(5):560-565. doi:http://dx.doi.org/10.1176/appi.ps.55.5.560.
62. Guo J, Konetzka RT, Manning WG. The causal effects of home care use on institutional long-term care utilization and expenditures. *Heal Econ (United Kingdom)*. 2015;24(S1):4-17. doi:http://dx.doi.org/10.1002/hec.3155.
63. Hadley J, Polsky D, Mandelblatt JS, et al. An exploratory instrumental variable analysis of the outcomes of localized breast cancer treatments in a medicare population. *Health Econ*. 2003;12(3):171-186. doi:http://dx.doi.org/10.1002/hec.710.
64. Hay JW, Lawler E, Yucel K, et al. Cost impact of diagnostic imaging for lower extremity peripheral vascular occlusive disease. *Value Heal*. 2009;12(2):262-266. doi:http://dx.doi.org/10.1111/j.1524-4733.2008.00438.x.
65. Huesch MD. External adjustment sensitivity analysis for unmeasured confounding: An application to coronary stent outcomes, Pennsylvania 2004-2008. *Health Serv Res*. 2013;48(3):1191-1214. doi:http://dx.doi.org/10.1111/1475-6773.12013.
66. Huybrechts KF, Brookhart MA, Rothman KJ, et al. Comparison of different approaches to confounding adjustment in a study on the association of antipsychotic medication with mortality in older nursing home patients. *Am J Epidemiol*. 2011;174(9):1089-1099. doi:http://dx.doi.org/10.1093/aje/kwr213.
67. Ionescu-Ittu R, Abrahamowicz M, Pilote L. Treatment effect estimates varied depending on the definition of the provider prescribing preference-based instrumental variables. *J Clin Epidemiol*. 2012;65(2):155-162. doi:http://dx.doi.org/10.1016/j.jclinepi.2011.06.012.
68. Kivimaki M, Vahtera J, Kawachi I, et al. Psychosocial work environment as a risk factor for absence with a psychiatric diagnosis: An instrumental-variables analysis. *Am J Epidemiol*. 2010;172(2):167-172. doi:http://dx.doi.org/10.1093/aje/kwq094.
69. Kramer A, Jager KJ, Fogarty DG, et al. Association between pre-transplant dialysis modality and patient and graft survival after kidney transplantation. *Nephrol Dial Transplant*. 2012;27(12):4473-4480. doi:http://dx.doi.org/10.1093/ndt/gfs450.
70. Kuo YF, Montie JE, Shahinian VB. Reducing bias in the assessment of treatment effectiveness: Androgen deprivation therapy for prostate cancer. *Med Care*. 2012;50(5):374-380. doi:http://dx.doi.org/10.1097/MLR.0b013e318245a086.
71. Lakdawalla DN, Mascarenhas M, Jena AB, et al. Impact of oral nutrition supplements on hospital outcomes in pediatric patients. *J Parenter Enter Nutr*. 2014;38(6):42S-49S. doi:http://dx.doi.org/10.1177/0148607114549769.
72. MacKenzie TA, Tosteson TD, Morden NE, Stukel TA, O’Malley AJ. Using instrumental variables to estimate a Cox’s proportional hazards regression subject to additive confounding. *Heal Serv Outcomes Res Methodol*. 2014;14(1-2):54-68. doi:http://dx.doi.org/10.1007/s10742-014-0117-x.
73. Margolis DJ, Gupta J, Hoffstad O, et al. Lack of effectiveness of hyperbaric oxygen therapy for the treatment of diabetic foot ulcer and the prevention of amputation a cohort study. *Diabetes Care*. 2013;36(7):1961-1966. doi:http://dx.doi.org/10.2337/dc12-2160.
74. Newman TB, Vittinghoff E, McCulloch CE. Efficacy of phototherapy for newborns with hyperbilirubinemia: a cautionary example of an instrumental variable analysis. *Med Decis Making*. 2012;32(1):83-92. http://ovidsp.ovid.com/ovidweb.cgi?T=JS&CSC=Y&NEWS=N&PAGE=fulltext&D=emed10&AN=21859678.
75. Parmar AD, Sheffield KM, Han Y, et al. Evaluating comparative effectiveness with observational data: Endoscopic ultrasound and survival in pancreatic cancer. *Cancer*. 2013;119(21):3861-3869. doi:http://dx.doi.org/10.1002/cncr.28295.
76. Pisoni RL, Arrington CJ, Albert JM, et al. Facility Hemodialysis Vascular Access Use and Mortality in Countries Participating in DOPPS: An Instrumental Variable Analysis. *Am J Kidney Dis*. 2009;53(3):475-491. doi:http://dx.doi.org/10.1053/j.ajkd.2008.10.043.
77. Prentice JC, Conlin PR, Gellad WF, Edelman D, Lee TA, Pizer SD. Capitalizing on prescribing pattern variation to compare medications for type 2 diabetes. *Value Heal*. 2014;17(8):854-862. doi:http://dx.doi.org/10.1016/j.jval.2014.08.2674.
78. Rassen JA, Brookhart MA, Glynn RJ, Mittleman MA, Schneeweiss S. Instrumental variables II: instrumental variable application-in 25 variations, the physician prescribing preference generally was strong and reduced covariate imbalance. *J Clin Epidemiol*. 2009;62(12):1233-1241. doi:http://dx.doi.org/10.1016/j.jclinepi.2008.12.006.
79. Rosenthal MB, Li Z, Robertson AD, Milstein A. Impact of financial incentives for prenatal care on birth outcomes and spending. *Health Serv Res*. 2009;44(5 Part 1):1465-1479. doi:http://dx.doi.org/10.1111/j.1475-6773.2009.00996.x.
80. Sheffield KM, Riall TS, Han Y, Kuo YF, Townsend, C. M. J, Goodwin JS. Association between cholecystectomy with vs without intraoperative cholangiography and risk of common duct injury. *Jama*. 2013;310(8):812-820. doi:http://dx.doi.org/10.1001/jama.2013.276205.
81. Steingrub JS, Lagu T, Rothberg MB, Nathanson BH, Raghunathan K, Lindenauer PK. Treatment with neuromuscular blocking agents and the risk of in-hospital mortality among mechanically ventilated patients with severe sepsis. *Crit Care Med*. 2014;42(1):90-96. doi:http://dx.doi.org/10.1097/CCM.0b013e31829eb7c9.
82. Stukel TA, Fisher ES, Wennberg DE, Alter DA, Gottlieb DJ, Vermeulen MJ. Analysis of observational studies in the presence of treatment selection bias: effects of invasive cardiac management on AMI survival using propensity score and instrumental variable methods. *JAMA*. 2007;297(3):278-285. doi:10.1001/jama.297.3.278.
83. Tagami T, Matsui H, Horiguchi H, Fushimi K, Yasunaga H. Antithrombin and mortality in severe pneumonia patients with sepsis-associated disseminated intravascular coagulation: An observational nationwide study. *J Thromb Haemost*. 2014;12(9):1470-1479. doi:http://dx.doi.org/10.1111/jth.12643.
84. VanDyke RD, McPhail GL, Huang B, et al. Inhaled tobramycin effectively reduces FEV1 decline in cystic fibrosis an instrumental variables analysis. *Ann Am Thorac Soc*. 2013;10(3):205-212. doi:http://dx.doi.org/10.1513/AnnalsATS.201209-082OC.
85. Wong K, Campitelli MA, Stukel TA, Kwong JC. Estimating influenza vaccine effectiveness in community-dwelling elderly patients using the instrumental variable analysis method. *Arch Intern Med*. 2012;172(6):484-491. doi:10.1001/archinternmed.2011.2038.
86. Bryson WC, McConnell J, Krothuis T, McCarty D. Extended-release naltrexone for alcohol dependence: persistence and healthcare costs and utilization. *Am J Manag Care*. 2011;17 Suppl 8:S222-234. internal-pdf://72.27.23.57/alcohol_depend-Bryson-AmJManCare11.pdf internal-pdf://4105231440/Bryson2011-suppl_appdx.docx.
87. Cheng L, Liu H, Zhang Y, Shen K, Zeng Y. The impact of health insurance on health outcomes and spending of the elderly: Evidence from china’s new cooperative medical scheme. *Heal Econ (United Kingdom)*. 2015;24(6):672-691. doi:http://dx.doi.org/10.1002/hec.3053.
88. De Preux LB. Anticipatory ex ante moral hazard and the effect of medicare on prevention. *Health Econ*. 2011;20(9):1056-1072. doi:http://dx.doi.org/10.1002/hec.1778.
89. Gebel M, Voßemer J. The impact of employment transitions on health in Germany. A difference-in-differences propensity score matching approach. *Soc Sci Med*. 2014;108:128-136. doi:10.1016/j.socscimed.2014.02.039.
90. Goetzel RZ, Roemer EC, Pei X, et al. Second-year results of an obesity prevention program at the dow chemical company. *J Occup Environ Med*. 2010;52(3):291-302. doi:http://dx.doi.org/10.1097/JOM.0b013e3181d46f0b.
91. Higgins S, Chawla R, Colombo C, Snyder R, Nigam S. Medical homes and cost and utilization among high-risk patients. *Am J Manag Care*. 2014;20(3):e61-e71. internal-pdf://67.10.22.98/medhomes_cost_highrisk-Higgins-AJMC14.pdf.
92. Kausto J, Viikari-Juntura E, Virta LJ, Gould R, Koskinen A, Solovieva S. Effectiveness of new legislation on partial sickness benefit on work participation: a quasi-experiment in Finland. *BMJ Open*. 2014;4(12):e006685. doi:http://dx.doi.org/10.1136/bmjopen-2014-006685.
93. Kelly Y, Kelly J, Sacker A. Changes in bedtime schedules and behavioral difficulties in 7 year old children. *Pediatrics*. 2013;132(5):e1184-e1193. doi:http://dx.doi.org/10.1542/peds.2013-1906.
94. Lin WC, Chien HL, Willis G, et al. The effect of a telephone-based health coaching disease management program on medicaid members with chronic conditions. *Med Care*. 2012;50(1):91-98. doi:http://dx.doi.org/10.1097/MLR.0b013e31822dcedf.
95. Lyon SM, Wunsch H, Asch DA, Carr BG, Kahn JM, Cooke CR. Use of intensive care services and associated hospital mortality after massachusetts healthcare reform. *Crit Care Med*. 2014;42(4):763-770. doi:http://dx.doi.org/10.1097/CCM.0000000000000044.
96. Menon J, Paulet M, Thomas IJ. Wellness coaching and health-related quality of life: A case-control difference-in-differences analysis. *J Occup Environ Med*. 2012;54(10):1259-1267. doi:http://dx.doi.org/10.1097/JOM.0b013e31825a2594.
97. Moran JR, Short PF, Hollenbeak CS. Long-term employment effects of surviving cancer. *J Health Econ*. 2011;30(3):505-514. doi:http://dx.doi.org/10.1016/j.jhealeco.2011.02.001.
98. Osborne NH, Nicholas LH, Ryan AM., Humma JR, Dimick JB. Association of hospital participation in a quality reporting program with surgical outcomes and expenditures for medicare beneficiaries. *JAMA - J Am Med Assoc*. 2015;313(5):496-504. doi:http://dx.doi.org/10.1001/jama.2015.25.
99. Rajaram R, Chung JW, Jones AT, et al. Association of the 2011 ACGME resident duty hour reform with general surgery patient outcomes and with resident examination performance. *JAMA - J Am Med Assoc*. 2014;312(22):2374-2384. doi:http://dx.doi.org/10.1001/jama.2014.15277.
100. Reid RO, Ashwood JS, Friedberg MW, Weber ES, Setodji CM, Mehrotra A. Retail clinic visits and receipt of primary care. *J Gen Intern Med*. 2013;28(4):504-512. doi:http://dx.doi.org/10.1007/s11606-012-2243-x.
101. Sadhu AR, Ang AC, Ingram-Drake LA, Martinez DS, Hsueh WA, Ettner SL. Economic benefits of intensive insulin therapy in critically Ill patients: The targeted insulin therapy to improve hospital outcomes (TRIUMPH) project. *Diabetes Care*. 2008;31(8):1556-1561. doi:http://dx.doi.org/10.2337/dc07-2456.
102. Sarkar U, Lyles CR, Parker MM, et al. Use of the refill function through an online patient portal is associated with improved adherence to statins in an integrated health system. *Med Care*. 2014;52(3):194-201. doi:http://dx.doi.org/10.1097/MLR.0000000000000069.
103. Watt C, Abuya T, Warren CE, Obare F, Kanya L, Bellows B. Can reproductive health voucher programs improve quality of postnatal care? A quasi-experimental evaluation of Kenya â€^TM^ s Safe Motherhood voucher scheme. *PLoS One*. 2015;10(4). doi:http://dx.doi.org/10.1371/journal.pone.0122828 April.
104. Domino ME, Norton EC, Morrissey JP, Thakur N. Cost shifting to jails after a change to managed mental health care. *Health Serv Res*. 2004;39(5):1379-1401. doi:http://dx.doi.org/10.1111/j.1475-6773.2004.00295.x.
105. Hodgkin D, Parks Thomas C, Simoni-Wastila L, Ritter GA, Lee S. The effect of a three-tier formulary on antidepressant utilization and expenditures. *J Ment Heal Policy Econ*. 2008;11(2):67-77. http://ovidsp.ovid.com/ovidweb.cgi?T=JS&CSC=Y&NEWS=N&PAGE=fulltext&D=med5&AN=18509214; http://lib.exeter.ac.uk:4556/resserv?sid=OVID:medline&id=pmid:18509214&id=doi:&issn=1091-4358&isbn=&volume=11&issue=2&spage=67&pages=67-77&date=2008&title=The+Journal+o.
106. Li J, Hurley J, DeCicca P, Buckley G. Physician response to pay-for-performance: evidence from a natural experiment. *Health Econ*. 2014;23(8):962-978. doi:http://dx.doi.org/10.1002/hec.2971.
107. Yoon J, Bernell SL. The role of adverse physical health events on the utilization of mental health services. *Health Serv Res*. 2013;48(1):175-194. doi:http://dx.doi.org/10.1111/j.1475-6773.2012.01442.x.
108. Tannen RL, Weiner MG, Xie D. Replicated studies of two randomized trials of angiotensin-converting enzyme inhibitors: Further empiric validation of the “prior event rate ratio” to adjust for unmeasured confounding by indication. *Pharmacoepidemiol Drug Saf*. 2008;17(7):671-685. doi:http://dx.doi.org/10.1002/pds.1584.
109. Tannen R, Weiner M, Xie D. Use of Primary Care Electronic Medical Record Database in Drug Efficacy Research on Cardiovascular Outcomes: Comparison of Database and Randomised. *BMJ Br Med J*. 2009;338(b81):1-9. http://www.jstor.org/stable/10.2307/20512072. Accessed January 9, 2014.
110. Tannen D.; Wang, X.; Yu, M.; Weiner, M. G. R. X. A new “Comparative Effectiveness” assessment strategy using the THIN database: Comparison of the cardiac complications of pioglitazone and rosiglitazone. *Pharmacoepidemiol Drug Saf*. 2013;22(1):86-97. doi:http://dx.doi.org/10.1002/pds.3360.
111. Brophy S, Jones KH, Rahman MA, et al. Incidence of Campylobacter and Salmonella Infections Following First Prescription for PPI: A Cohort Study Using Routine Data. *Am J Gastroenterol*. April 2013. doi:10.1038/ajg.2013.30.
112. Uddin MJ, Groenwold RHH, van Staa TP, et al. Performance of prior event rate ratio adjustment method in pharmacoepidemiology: a simulation study. *Pharmacoepidemiol Drug Saf*. November 2014. doi:10.1002/pds.3724.
113. Albouy V, Lequien L. Does compulsory education lower mortality? *J Health Econ*. 2009;28(1):155-168. doi:http://dx.doi.org/10.1016/j.jhealeco.2008.09.003.
114. Swaminathan S, Mor V, Mehrotra R, Trivedi AN. Effect of medicare dialysis payment reform on use of erythropoiesis stimulating agents. *Health Serv Res*. 2015;50(3):790-808. doi:http://dx.doi.org/10.1111/1475-6773.12252.
115. Zuckerman IH, Lee E, Wutoh AK, Xue Z, Stuart B. Application of regression-discontinuity analysis in pharmaceutical health services research. *Health Serv Res*. 2006;41(2):550-563. doi:10.1111/j.1475-6773.2005.00487.x.
116. Wagner TH, Jimison HB. Computerized health information and the demand for medical care. *Value Heal*. 2003;6(1):29-39. doi:http://dx.doi.org/10.1046/j.1524-4733.2003.00155.x.
117. Fortney JC, Steffick DE, Burgess Jr JF, Maciejewski ML, Petersen LA. Are primary care services a substitute or complement for specialty and inpatient services? *Health Serv Res*. 2005;40(5):1422-1442. doi:http://dx.doi.org/10.1111/j.1475-6773.2005.00424.x.
118. Chung S, Domino ME, Stearns SC. The effect of retirement on weight. *journals Gerontol*. 2009;Series B,(5):656-665. http://ovidsp.ovid.com/ovidweb.cgi?T=JS&CSC=Y&NEWS=N&PAGE=fulltext&D=emed9&AN=19357073; http://lib.exeter.ac.uk:4556/resserv?sid=OVID:embase&id=pmid:19357073&id=doi:&issn=1758-5368&isbn=&volume=64&issue=5&spage=656&pages=656-665&date=2009&title=The+journa.
119. Hay J, Jhaveri M, Tangirala M, Kaliner M. Cost and resource utilization comparisons of second-generation antihistamines vs. montelukast for allergic rhinitis treatment. *Allergy Asthma Proc*. 2009;30(6):634-642. doi:http://dx.doi.org/10.2500/aap.2009.30.3293.
120. Kawatkar AA, Hay JW, Stohl W, Nichol MB. Incremental expenditure of biologic disease modifying antirheumatic treatment using instrumental variables in panel data. *Heal Econ (United Kingdom)*. 2013;22(7):807-823. doi:http://dx.doi.org/10.1002/hec.2855.
121. Piernas C, Ng SW, Mendez MA, Gordon-Larsen P, Popkin BM. A dynamic panel model of the associations of sweetened beverage purchases with dietary quality and food-purchasing patterns. *Am J Epidemiol*. 2015;181:661-671. doi:http://dx.doi.org/10.1093/aje/kwu317.
